# *SLC4A1* mutations that cause distal renal tubular acidosis alter cytoplasmic pH and cellular autophagy

**DOI:** 10.7554/eLife.108253

**Published:** 2026-03-04

**Authors:** Grace Essuman, Midhat Rizvi, Ensaf Almomani, Shahid AKM Ullah, Sarder MA Hasib, Forough Chelangarimiyandoab, Priyanka Mungara, Manfred J Schmitt, Marguerite Hureaux, Rosa Vargas-Poussou, Nicolas Touret, Emmanuelle Cordat

**Affiliations:** 1 Department of Physiology, University of Alberta Edmonton Canada; 2 Department of Basic Medical Sciences, Faculty of Medicine, Al-Balqa Applied University Al-Salt Jordan; 3 Department of Medicine, University of Alberta Edmonton Canada; 4 Department of Molecular and Cell Biology, Department of Biosciences (FR 8.3) and Center of Human and Molecular Biology (ZHMB), Saarland University Saarbrücken Germany; 5 Department of Genetics, Georges Pompidou European Hospital Paris France; 6 Department of Biochemistry, University of Alberta Edmonton Canada; Oregon Health and Science University United States; University of Cambridge United Kingdom

**Keywords:** mouse, transgenic animals, kidney, Mouse

## Abstract

Distal renal tubular acidosis (dRTA) is a disorder characterized by the inability of the collecting duct system to secrete acids during metabolic acidosis. The pathophysiology of dominant or recessive *SLC4A1* variant-related dRTA has been linked with the mis-trafficking defect of mutant kAE1 protein. However, in vivo studies in kAE1 R607H dRTA mice and humans have revealed a complex pathophysiology implicating a loss of kAE1-expressing intercalated cells and intracellular relocation of the H^+^-ATPase in the remaining type-A intercalated cells. These cells also displayed accumulation of ubiquitin and p62 autophagy markers. The highly active transport properties of collecting duct cells require the maintenance of cellular energy and homeostasis, a process dependent on intracellular pH. Therefore, we hypothesized that the expression of dRTA variants affects intracellular pH and autophagy pathways. In this study, we report the characterization of newly identified dRTA variants and provide evidence of abnormal autophagy and degradative pathways in mouse inner medullary collecting duct cells and kidneys from mice expressing kAE1 R607H dRTA mutant protein. We show that reduced transport activity of the kAE1 variants correlated with increased cytosolic pH, reduced ATP synthesis, attenuated downstream autophagic pathways pertaining to the fusion of autophagosomes and lysosomes and/or lysosomal degradative activity. Our study elucidated a close relationship between the expression of defective kAE1 proteins, reduced mitochondrial activity, and decreased autophagy and protein degradative flux.

## Introduction

Distal renal tubular acidosis (dRTA) is a disorder characterized by the inability of the collecting duct system to secrete acids during metabolic acidosis (Giglio et al., 2021). In addition to hyperchloremic metabolic acidosis, patients with this disease can present with hypokalemia, kidney stones, urinary sodium waste, and difficulty thriving. Expression of pathogenic variants in the *ATP6V0A4*, *ATP6V1B1*, *FOXI1*, *WDR72,* and *SLC4A1* genes are the usual genetic etiologies (Escobar et al., 2016; Rungroj et al., 2018; Enerbäck et al., 2018). The *SLC4A1* gene encodes the anion exchanger 1 (AE1) protein, which is an electroneutral chloride/bicarbonate exchanger (Toye et al., 2004). It exists in two forms: a 911 amino acid erythroid isoform known to interact with erythroid cytoskeletal proteins and participate in red cell respiration and integrity, and a 65 amino acid (NH_2_-terminal) truncated isoform primarily found in the basolateral membrane of renal type A intercalated cells (A-IC) (Kollert-Jons et al., 1993; Nuiplot et al., 2015) and podocytes (Wu et al., 2010). This isoform participates in bicarbonate reabsorption and through its physical and functional interaction with the cytosolic carbonic anhydrase II and apical H^+^-ATPase, it supports apical proton export and urine acidification (Cordat and Reithmeier, 2014).

Pathogenic variants in the *SLC4A1* gene can result in either red cell defects (such as Southeast Asian ovalocytosis [Sawasdee et al., 2006; Cheung et al., 2005] and hereditary spherocytosis [Tang et al., 2020]), renal cell defects (dRTA; Sawasdee et al., 2006) or both in patients with homozygous (Band 3 Coimbra and Band 3 Courcouronnes; Ribeiro et al., 2000; Toye et al., 2008) or compound heterozygous variants (Chang et al., 2009; Khositseth et al., 2012). Renal *SLC4A1* disease-causing variants have only been found in the transmembrane domain—where it could impact protein structure and its transport function—or in the short carboxyl domain—where it possibly affects protein–protein interactions. The pathophysiology of dominant or recessive *SLC4A1*-related dRTA (hereafter named dRTA) has originally been linked with the mis-trafficking defect of mutant kAE1 protein (Sawasdee et al., 2006; Cordat, 2006). However, recent in vivo studies in R607H (orthologous to human R589H dRTA variant) and L919X knockin mice and dRTA patients have revealed a complex pathophysiology where kAE1-expressing intercalated cells were lost, and in the remaining type-A intercalated cells, the H^+^-ATPase relocated intracellularly and accumulated autophagy marker p62 and ubiquitin-positive material (Mumtaz et al., 2017).

The highly active transport properties of collecting duct cells require the tight maintenance of cellular energy and homeostasis. The autophagy-mediated turnover of damaged organelles is necessary for protecting collecting duct cells as in most renal cells (Festa et al., 2018). The chloride/bicarbonate exchange function of kAE1 in A-ICs confers a pivotal role in pH homeostasis and thus is a major contributor to cellular homeostasis. kAE1 protein interacts with several proteins such as integrin-like kinase (ILK), adaptor-related protein complex 1, 3, and 4 (AP-1, AP-3, and AP-4 mu1A), transmembrane protein 139 (TMEM139), kinesin family member 3B (KIF3B), and clathrin, among others (Nuiplot et al., 2015; Keskanokwong et al., 2007; Duangtum et al., 2011; Sawasdee et al., 2010) that support protein stability and trafficking. It also interacts with homeostatic proteins including the glycolytic enzyme glyceraldehyde-3-phosphate dehydrogenase (GAPDH) Su et al., 2011 and the antioxidant enzyme peroxiredoxin 6 (PRDX 6) (Sorrell et al., 2016), which play major roles in cellular energy metabolism and oxidative stress response, respectively.

In this study, we report the characterization of newly identified dRTA genetic variations and provide evidence of abnormal autophagy and degradative pathways in cells and kidneys from mice expressing dRTA mutant kAE1 proteins.

## Results

### The dRTA kAE1 variants traffic to the basolateral membrane but have reduced transport activity in mIMCD3 cells

**Figure 1.**
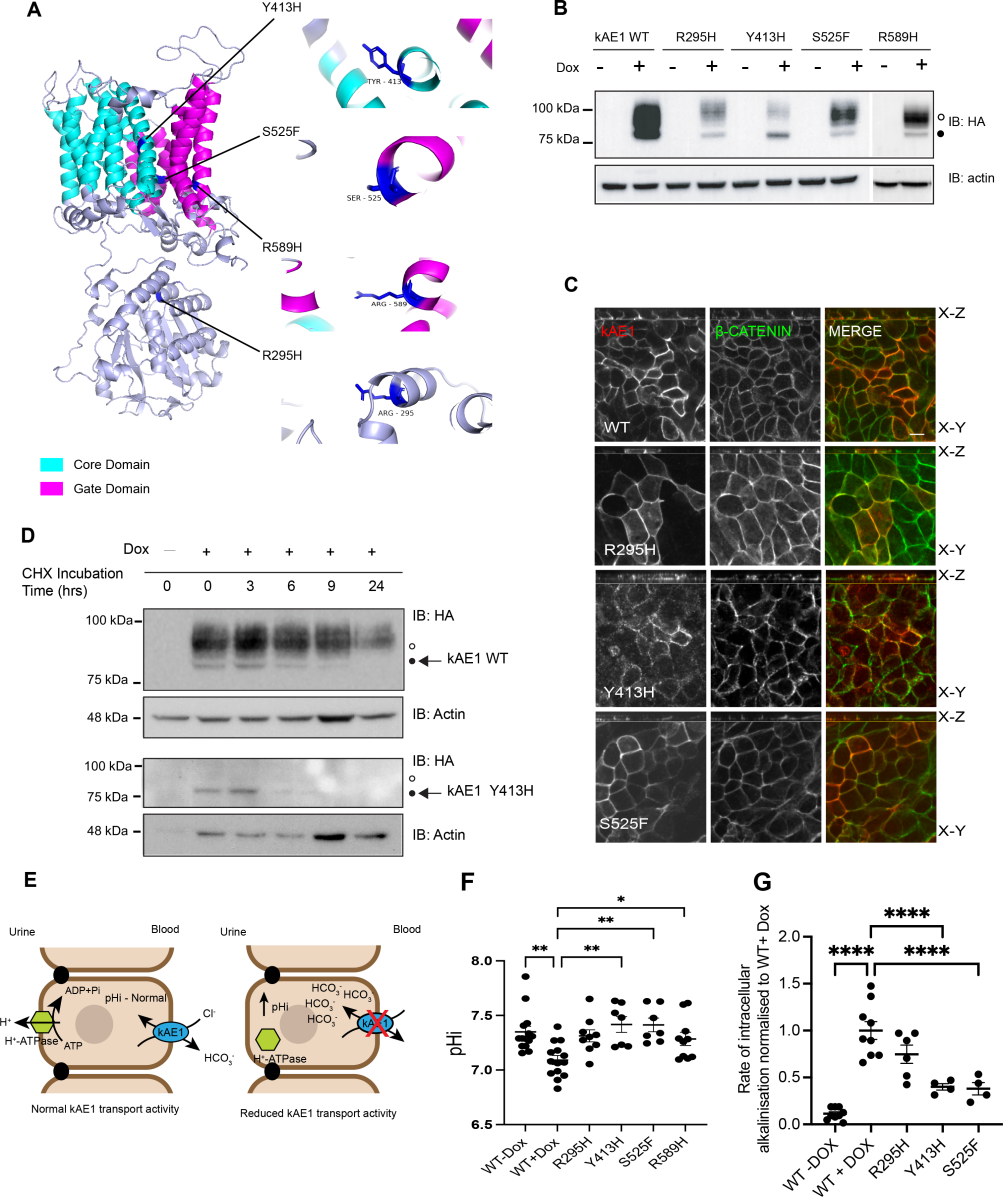
kAE1 R295H, Y413H, S525F, and R589H dRTA mutants are either dysfunctional or prematurely degraded. (**A**) Alpha Fold predicted structure of the kidney isoform of Band 3 anion exchanger 1 (kAE1) with core and gate domains highlighted. The dRTA kAE1 mutation sites are colored in blue with line extensions detailing specific amino acids mutated. (**B**) Immunoblot showing expression of kAE1 WT, R295H, Y413H, S525F, and R589H and corresponding actin band in mIMCD3 cells treated with and without doxycycline for 24 hours. Mouse anti-HA antibody was used to detect kAE1-HA, top (open circle) and bottom bands (closed circle) correspond to kAE1 carrying complex and high mannose oligosaccharides, respectively. (**C**) Immunostaining of kAE1 WT or mutant (red) and β-catenin (green) in polarized mIMCD3 cells. Scale bar = 10 μm. Red = kAE1, green = ß-catenin. (**D**) Immunoblot of cycloheximide (CHX) chase assay with corresponding actin in kAE1 mIMCD3 WT and Y413H cells showing the degradation of kAE1 Y413H after 3 hours CHX incubation. (**E**) Cartoon depicting the transporter activity and expected changes in pHi in cells expressing kAE1 WT (left) or inactive mutant (right). (**F**) Graphical representation of intracellular pH (pHi) measurement of mIMCD3 kAE1 WT, R295H, Y413H, S525F, and R589H cells. Error bars correspond to mean ± SEM, n=minimum 32. *p<0.05, **p<0.01 using one-way ANOVA followed by a Dunnett’s post hoc test. (**G**) Rate of intracellular alkalinization in WT or mutant mIMCD3 cells normalized to WT + Dox. **** indicates p<0.0001 using one-way ANOVA followed by a Dunnett’s post hoc test. Error bars correspond to mean ± SEM, n=minimum 4.

We first characterized three newly identified dRTA mutations and compared them with kAE1 WT or previously characterized kAE1 R589H mutant. Figure 1A depicts the alpha fold predicted structure of kAE1 showing amino acids mutated in each kAE1 mutant. kAE1 R295H is a recessively inherited substitution in the N-terminal cytosolic domain of the protein. kAE1 Y413H is a dominantly inherited substitution in transmembrane domain (TM) 1 of the core domain. In the gate domain, dominantly inherited S525F and R589H substitutions occur in TM 5 and TM 6, respectively. We generated the newly identified dRTA variant cDNAs and expressed them or kAE1 WT in mIMCD3 cells. As seen on Figure 1B, kAE1 R295H, S525F, and R589H variants display two typical bands similar to kAE1 WT. The top band (open circle) encompasses kAE1 proteins carrying a complex oligosaccharide that have reached the Golgi and beyond, while the bottom band (black circle) corresponds to high mannose-carrying kAE1 proteins located in the endoplasmic reticulum. However, kAE1 Y413H mutant bands intensity was overall weaker than WT and displayed a predominant single band aligned with high mannose-carrying kAE1 proteins. These results indicate that the three newly described dRTA mutants are successfully expressed in mIMCD3 cells. We next localized these mutants by immunofluorescence in polarized mIMCD3 cells. Both kAE1 WT and mutants appropriately co-localized with basolateral membrane marker beta-catenin in polarized mIMCD3 cells (Figure 1C). kAE1 R589H location has previously been reported at the basolateral membrane in polarized mIMCD3 cells (Cordat, 2006). However, staining for kAE1 Y413H was again weaker and seemed more intracellular than other mutants. To address a possible premature degradation of this variant, we measured its lifetime and observed that its degradation begins 6 hours post-synthesis while kAE1 WT abundance remained stable for 24 hours (Figure 1D). Finally, to assess the transport activity of the mutants, we examined the steady-state cytosolic pH (pHi) and rate of intracellular alkalinization of mIMCD3 cells expressing kAE1 WT or mutants (Figure 1E–G). Using BCECF-AM, we observed that the steady state intracellular pH (pHi) of kAE1 mutant cells was more alkaline than WT cells (except for kAE1 R295H cell pHi, which has a similar trend but is not significantly different from WT) (Figure 1F), in agreement with rate of intracellular alkalinization (Figure 1G). Note that transport activity, measured as rate of alkalinization, is measured by reverting kAE1 exchange activity from bicarbonate *export* to *import* (see ‘Materials and methods, protocol for transport assay), hence a reduced alkalinization rate is observed in cells expressing defective kAE1 protein compared to WT (Figure 1G). Overall, these results indicate that except for the kAE1 Y413H mutant, the other newly described variants are expressed and traffic to the basolateral membrane of polarized mIMCD3 cells, similar to the previously published kAE1 R589H mutant. Given that the premature degradation of kAE1 Y413H mutant likely explains the dRTA phenotype, we did not perform further assays on cells expressing this protein.

### Autophagy processes are altered in mIMC3 cells expressing the kAE1 R295H, S525F, and R589H dRTA mutants and in R607H knock-in kidney lysates

**Figure 2.**
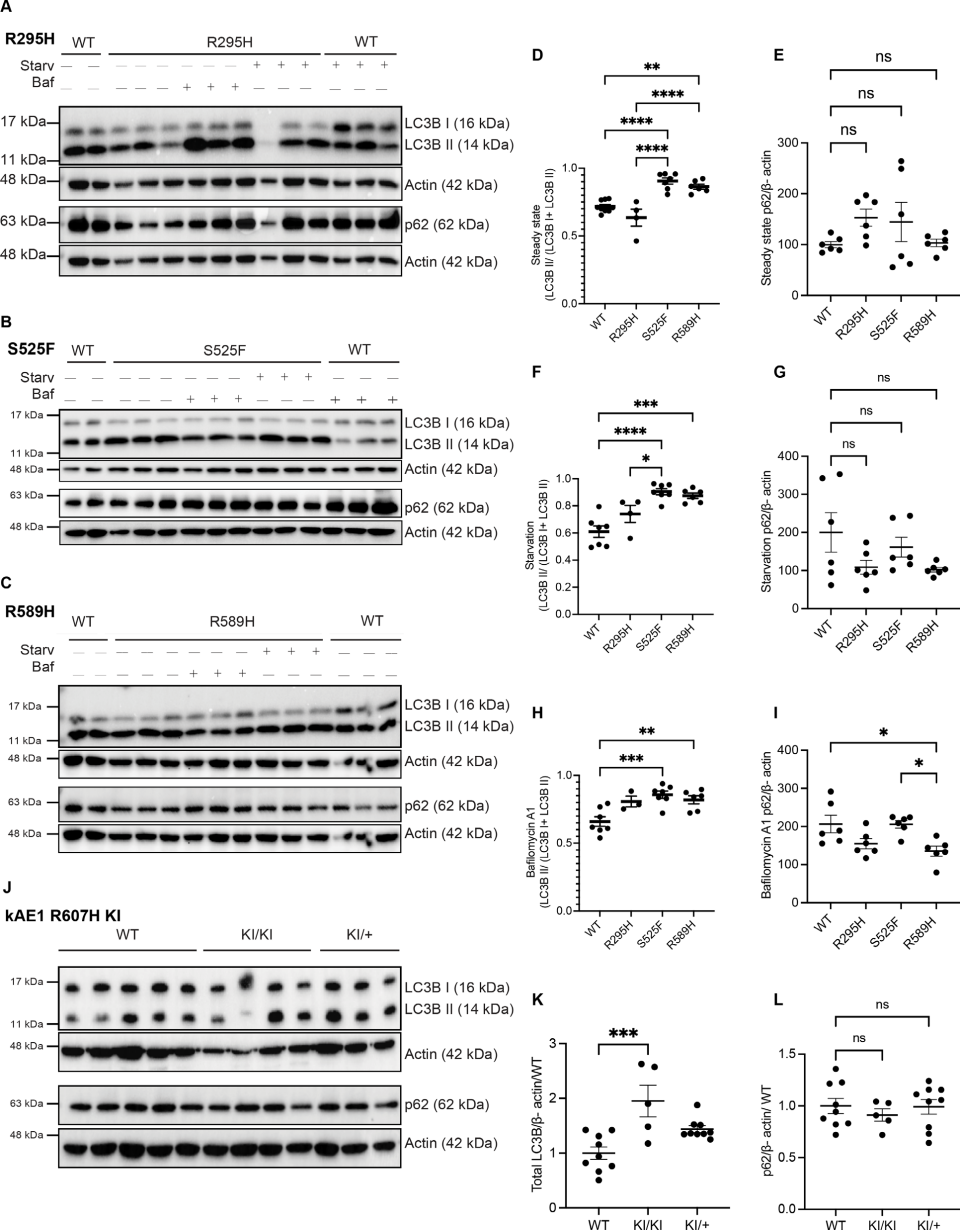
Autophagy is upregulated in dRTA kAE1 mutants in vitro and in vivo. . (**A–C**) Representative immunoblots of LC3B and p62 with corresponding actin abundance in kAE1 WT, R295H, S525F, and R589H mIMCD3 cells at steady state, under starvation (Starv) or 400 nM Bafilomycin A1 (Baf) treatment. Note that p62 and LC3B were detected on the same blot for (A) and (C); therefore, the same actin blot is shown for both panels. (**D–I**) Quantification of all immunoblots showing the ratio of LC3B II to total LC3B and p62. Error bars correspond to mean ± SEM, n=3–8. *p<0.05, ** p<0.01, ***p<0.005, ****p<0.001 using one-way ANOVA followed by a Tukey’s post hoc test. Immunoblots (**J**) and quantification (**K, L**) of LC3B and p62 abundance in kAE1 R607H KI mouse whole kidney lysates. Error bars correspond to mean ± SEM, n=minimum 5. ***p<0.005 using one-way ANOVA followed by a Tukey’s post hoc test.

In mice expressing kAE1 R607H (the murine equivalent to human dRTA R589H substitution), a striking reduction in type-A intercalated cells was noted, and in the remaining cells, autophagy marker p62 and ubiquitin accumulated in these abnormally enlarged cells (Mumtaz et al., 2017). We therefore investigated the autophagy machinery in dRTA mutant mIMCD3 cells and in R607H knock-in (KI) mice. We first examined the ratio of autophagosome marker LC3BII protein relative to the total intensities of LC3BI and LC3BII as well as p62 levels. These experiments were performed at steady state, upon autophagy induction by starvation, or after autophagy inhibition by Bafilomycin (Baf) A1 (Figure 2A–C). Figure 2A–I shows a consistent increase in the ratio of LC3B II to total LC3B (I+II) in the mutant cells at steady state (Figure 2D), with starvation (Figure 2F) and with Baf A1 (Figure 2H) except for the kAE1 R295H mutant which was not significantly different from WT. This suggests an altered autophagy process in the mutant cells, in agreement with preliminary findings from Mumtaz and colleagues (Mumtaz et al., 2017). There was no significant difference in p62 abundance in mutant cells compared to WT at steady state and with starvation (Figure 2E and G). However, with Baf A1, R589H mutant cells had significantly lower p62 abundance compared to WT (Figure 2I). To confirm these findings, we next assessed the abundance of these markers in whole kidney lysates from the kAE1 R607H KI mice. We observed that the total LC3B abundance was significantly higher in homozygous KI mice (KI/KI) compared to WT, with no difference in p62 (Figure 2J–L). Overall, these results suggest an abnormal autophagy in mutant mIMCD3 cells and in kidneys of R607H KI mice. Given the lack of difference in phenotype between the recessive kAE1 R295H and kAE1 WT mIMCD3 cells, we focused the subsequent experiments on dominant kAE1 S525F and R589H mutant cells. Further investigations will be needed to understand the pathophysiology associated with the kAE1 R295H novel variant.

### Late autophagy steps are blocked in dRTA kAE1 mutant-expressing cells due to their alkaline intracellular pH

**Figure 3.**
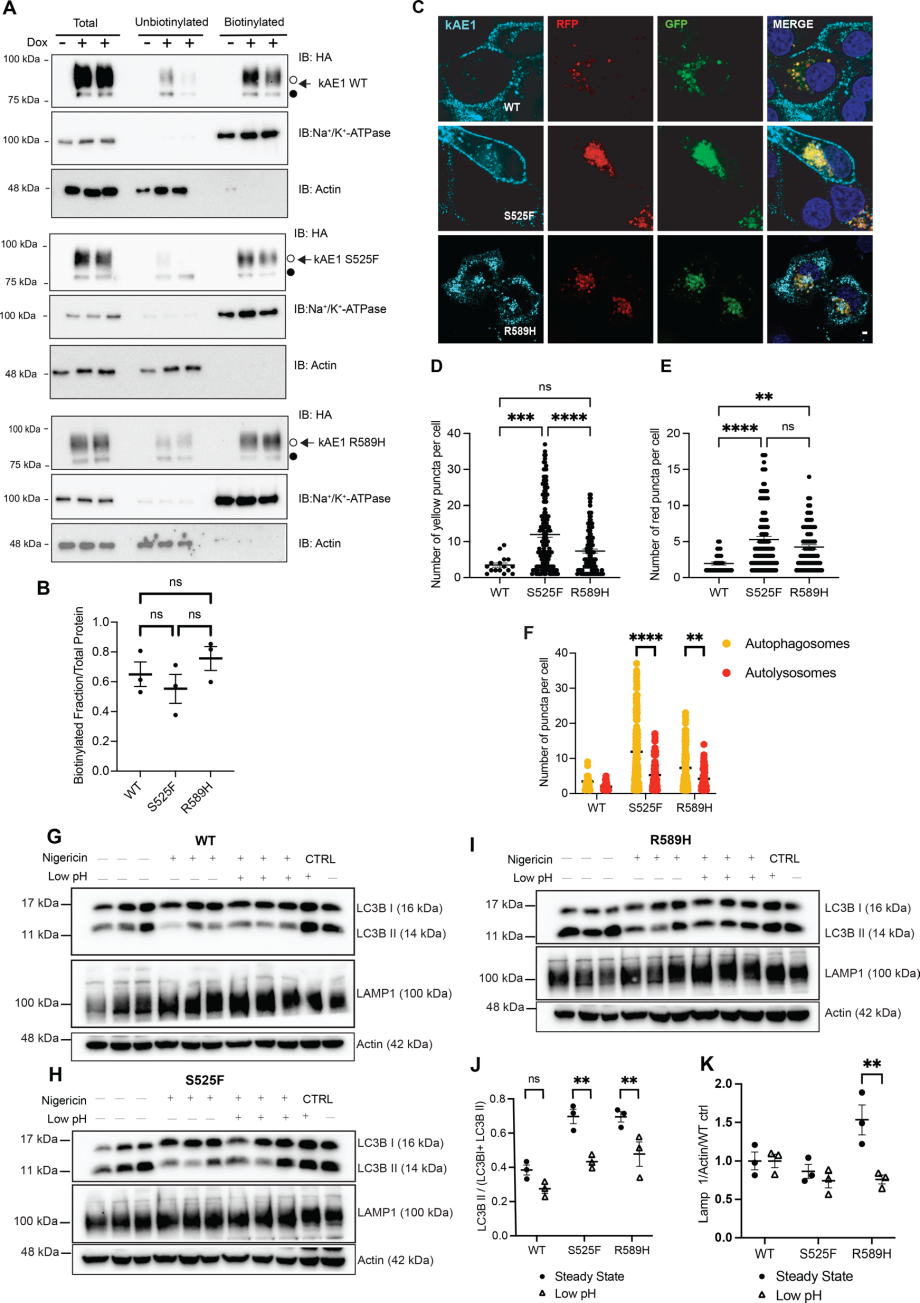
dRTA kAE1 mutants have more alkaline steady-state intracellular pH and altered autophagy flux. (**A**) Representative immunoblots of cell surface biotinylation experiments in mIMCD3 cells expressing kAE1 WT or mutants (top panels), with control staining of plasma membrane marker Na^+^/K^+^-ATPase (middle panel) and intracellular marker actin (bottom panel). (**B**) Quantification of three independent cell surface biotinylation experiments. Data are represented by a single representative blot for each variant. n.s., not significant using one-way ANOVA. Error bars correspond to mean ± SEM, n=3. (**C**) Immunofluorescence staining in eGFP-RFP-LC3 transfected mIMCD3 cells expressing kAE1. GFP = green, RFP = red, kAE1=cyan, nuclei = dark blue (merge only). Scale bar = 2 μm. Graphical representation of number of yellow (autophagosomes) (**D**) and red (autolysosomes) (**E**) puncta per cell expressing kAE1. Error bars correspond to mean ± SEM, n=minimum 32. **p<0.01, *** p<0.005, ****p<0.001 using one-way ANOVA followed by a Tukey’s post hoc test. (**F**) Grouped graph of the number of yellow (autophagosomes) and red (autolysosomes) puncta per cell expressing kAE1, respectively. Note that the statistical analysis displayed only compared yellow and red groups for simplification. Error bars correspond to mean ± SEM, n=minimum 32. **p<0.01, ****p<0.001 using two-way ANOVA followed by a Sidak’s post hoc test. (**G–I**) Immunoblot of LC3B, LAMP1, and actin in kAE1 WT, S525F, and R589H mIMCD3 cells at steady state and under chemically reduced intracellular pH conditions. Graphical representation of the ratio of LC3B II to total LC3B ratio (**J**) or LAMP1 (**K**) at steady state versus at low pHi in mIMCD3 kAE1 WT, S525F, and R589H. Black circles indicate steady state cells and triangles indicate low pHi cells. Error bars correspond to mean ± SEM, n=3. ** indicates p<0.01 using two-way ANOVA followed by a Sidak’s post hoc test.

Given these preliminary findings of abnormal autophagy in dRTA mutant cells, we examined in more detail their autophagy machinery by transiently transfecting them with the eGFP-RFP-LC3 construct and monitoring the rate of autophagosome and autolysosome formation. In this assay, the green fluorescent protein (eGFP) fused to LC3 is quenched in the acidic environment of the autolysosome, while both eGFP and red fluorescent protein (RFP) fluoresce in vesicles in the neutral lumen of the autophagosome (Zhou et al., 2012; Kimura et al., 2007). To avoid the poor efficiency of transient transfection in polarized cells, experiments were conducted in sub-confluent cells, pending that the mutant proteins are present at the plasma membrane. We therefore performed cell surface biotinylations on 70–80% sub-confluent cells, which confirmed a robust plasma membrane abundance of both kAE1 R589H and S525F that was not significantly different from the WT protein (Figure 3A and B). Therefore, we next assessed the efficiency of the autophagy machinery in WT or dRTA mutant-expressing cells (Figure 3C–F). Focusing on cells expressing either kAE1 WT, kAE1 S525F, or R589H, we quantified the number RFP+ (red, acidic autolysosomes) and double eGFP+/RFP+ (yellow, not acidic autophagosomes) vesicles. kAE1 S525F mutant cells have significantly more autophagosomes than WT counterparts (Figure 3D)**,** and both kAE1 S525F and R589H have significantly more autolysosomes than WT (Figure 3E). Figure 3F shows that both mutants had significantly more autophagosomes (yellow) than autolysosomes (red). This finding suggests an upregulation of autophagy and inhibition in the downstream steps of the process that involves the fusion of autophagosomes with the lysosome (Mizushima, 2018). As autolysosomes require luminal v-H^+^-ATPase-dependent acidification to efficiently clear cell debris (Hu et al., 2022), the higher intracellular pH seen in mutant cells (Figure 1F) may be detrimental to v-H^+^-ATPase full activity and impair proper autolysosomal acidification (Berezhnov et al., 2016). We therefore wondered whether chemically acidifying the pHi in mutant cells would rescue the autophagy machinery (Figure 3G–K). We first determined that incubation of mIMCD3 cells in 0.033 µM nigericin in cell culture medium at pH 6.6 for 2 hours acidified cytosolic pH to 6.9 without causing cell death (Figure 3—figure supplement 1). Next, we observed that chemically reducing pHi to 6.9 in mutant expressing cells reduced the ratio of LC3B II to total LC3B (Figure 3J) and the abundance of lysosomal-associated membrane protein 1 (LAMP1) in R589H cells (Figure 3K) to levels similar to WT cells at steady state. These findings suggest that abnormal autophagy in the mutant cells may be caused by their alkaline pHi, resulting from a reduced anion exchange activity of the mutant kAE1 protein.

**Figure 3—figure supplement 1.**
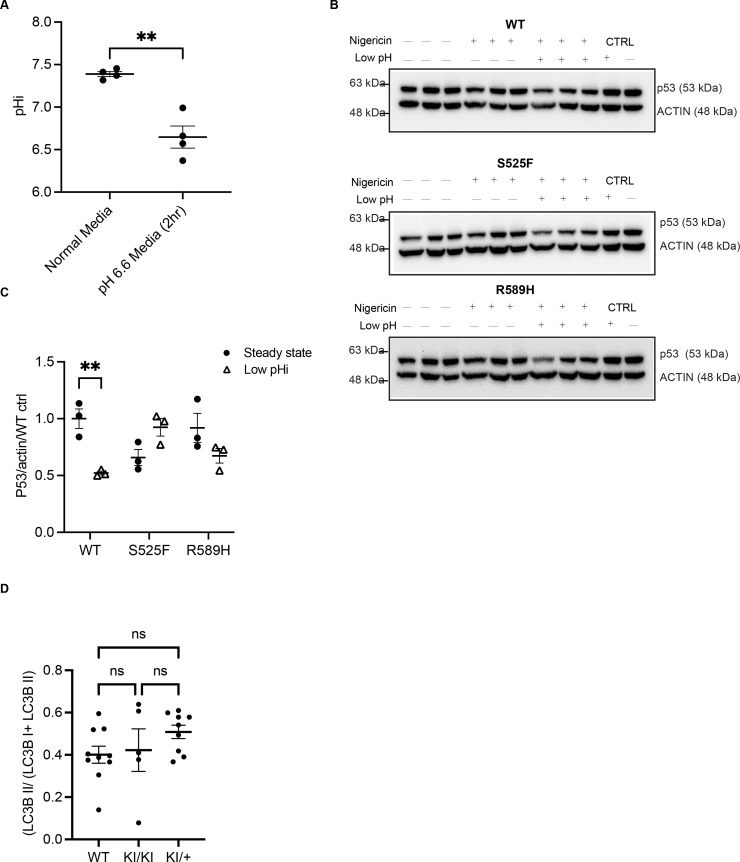
Two-hour incubation in pH 6.6 media with 0.033 µM nigericin reduces cytosolic pH to 6.9. (**A**) pHi of mIMCD3 cells incubated in either normal pH media of pH 6.6 media with 0.033 µM nigericin. Error bars correspond to mean ± SEM, n = minimum 4. ** *p*<0.01, using Student’s t test. (**B**) Immunoblots of p53 with corresponding actin abundance in mIMCD3 cells at steady state and under chemically reduced intracellular pH conditions. (**C**) Graphical representation of ratio of p53 at steady state versus at low pHi in mIMCD3 kAE1 WT, S525F and R589H cells. Black circles indicate steady state cells and triangles indicate low pHi cells. Error bars correspond to mean ± SEM, n = 3. ** indicate *p*<0.01 using two-way ANOVA.

### mIMCD3 cells expressing dRTA kAE1 mutants and R607H KI kidney tissues have abnormal lysosome number and size

**Figure 4.**
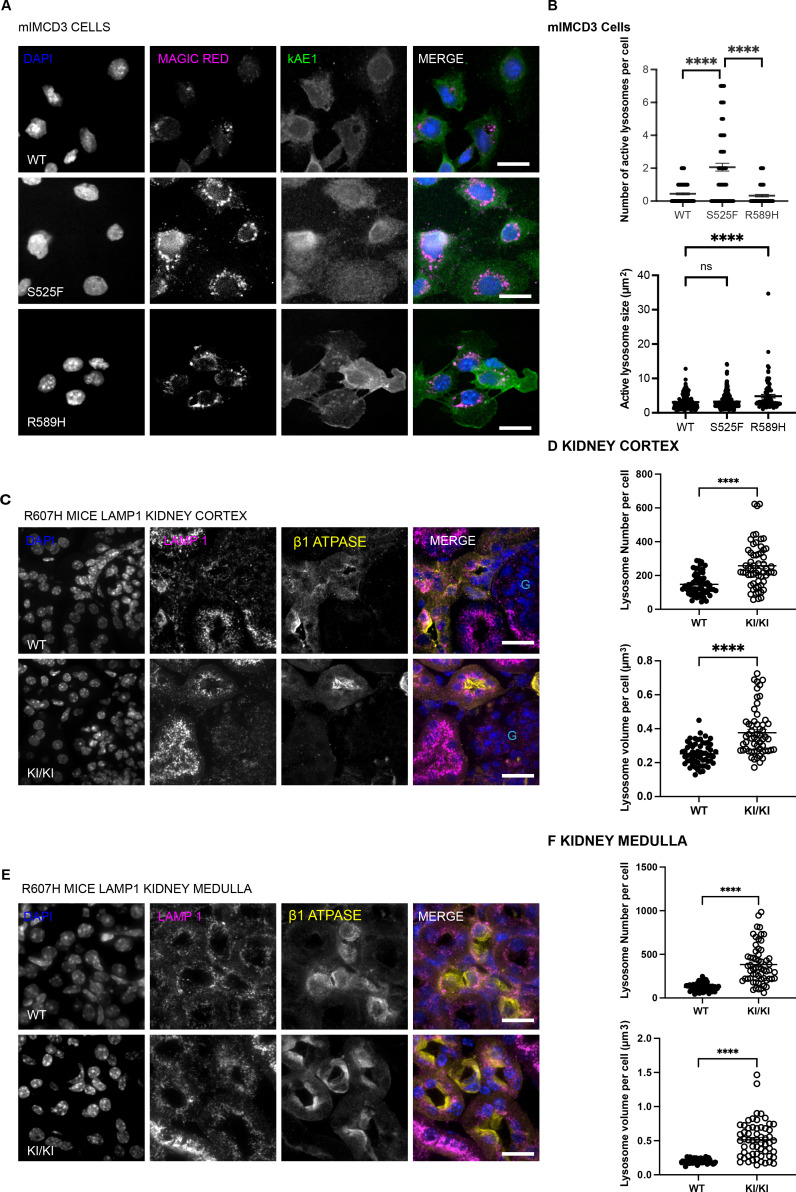
dRTA kAE1 mutants have bigger or more lysosomes than WT in vitro and in vivo. (**A**) Immunofluorescence images of kAE1 WT, S525F, and R589H mIMCD3 cells incubated with Magic Red substrate for 1 hour at 37°C in the dark. Green = kAE1, magenta = active lysosomes, blue = nuclei. Scale bar = 16 µm. (**B**) Graphical representation of number and size of active lysosomes per cell. Error bars correspond to mean ± SEM, n=minimum 30. ****p<0.0001 using one-way ANOVA followed by Tukey’s post hoc test. Immunofluorescence images of LAMP1 and ß1 ATPase in kidney cortex (**C**) or medulla (**E**) from kAE1 WT and R607H KI mice. Blue = nuclei, magenta = LAMP1 (lysosomes), yellow = ß1 ATPase, light blue ‘G’ indicates the location of a glomerulus. Scale bar = 8 μm. Graphical representation of the number and volume of LAMP1 vesicles in ß1 ATPase-positive cells in the kidney cortex (**D**) or medulla (**F**) of WT or R607H KI mice. Error bars correspond to mean ± SEM, n=60. ****p<0.001 using Student’s *t*-test.

The accumulation of autophagosomes and autolysosomes as seen above may suggest one or a combination of the following: an inability of autophagosomes to fuse with lysosomes and/or a defect in lysosomal degradative activity in the mutant cells (Button et al., 2017; Festa et al., 2018). We first examined the lysosomal degradative activity by assessing lysosomal protease Cathepsin B activity using Magic Red staining, a probe that fluoresces upon lysosomal protease cleavage (Figure 4A and B). In agreement with increased RFP+ vesicles (Figure 3E), the kAE1 S525F mutant cells had a significantly higher number of Magic Red-positive vesicles than WT, whereas the kAE1 R589H mutant cells had significantly larger Magic Red-positive vesicles, suggesting an accumulation of undigested material (de Araujo et al., 2020). To validate this finding in vivo, we performed immunostaining and quantified LAMP1-positive staining in ß1 ATPase-positive cells (a marker of ICs) in WT and R607H KI mouse kidney sections. The KI mice showed significantly more and larger LAMP1-positive vesicles compared to WT mice in both cortex and medulla (Figure 4C–F). We probed further into the lysosomal activity by quantifying lysosomal protease Cathepsin D (immature, intermediate, and mature) protein abundance by immunoblot in isolated primary murine A-ICs. Although the abundance of immature and intermediate cathepsin D did not differ between genotypes, the KI mice showed a significantly decreased abundance of mature cathepsin D (Figure 4—figure supplement 1). Thus, in line with in vitro findings, A-IC from homozygous R607H KI mice display relatively more and larger lysosomes with reduced active protease abundance than WT littermates, suggesting a lysosomal defect in the dRTA kAE1 mutant cells.

**Figure 4—figure supplement 1.**
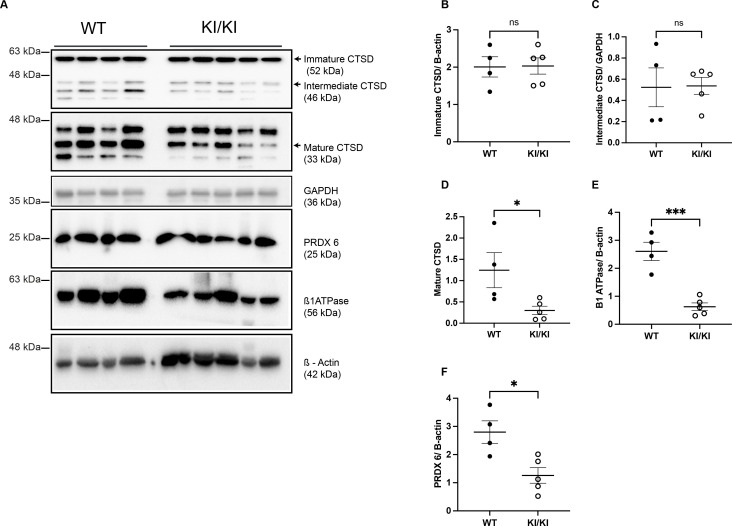
dRTA kAE1 R607H knockin mice have lower abundance of intercalated cell marker B1 H+-ATPase, lysosomal protease Cathepsin D and antioxidant protein PRDX 6 in intercalated cells-enriched primary cells. (**A**) Immunoblot of primary cells showing protein abundance of immature, intermediate and mature Cathepsin D (CTSD), antioxidant protein PRDX6 and intercalated cell marker B1 H+-ATPase with corresponding GAPDH and actin abundance. The first two panels are from the same blot but the second one is cropped on the region around 48 kDa and lower, and exposed longer. Primary cells were obtained by magnetic sorting and lysed upon collection. (**B-F**) Graphical representation of protein abundance of immature CTSD, intermediate CTSD, mature CTSD, B1 H+-ATPase and PRDX6 respectively in WT and knockin intercalated cell-enriched primary cells. Error bars correspond to mean ± SEM, n = minimum 4. * *p*<0.05, *** *p*<0.005 using Student’s t test.

### dRTA kAE1 mutant cells have lower ATP production rate and abnormal mitochondrial content

**Figure 5.**
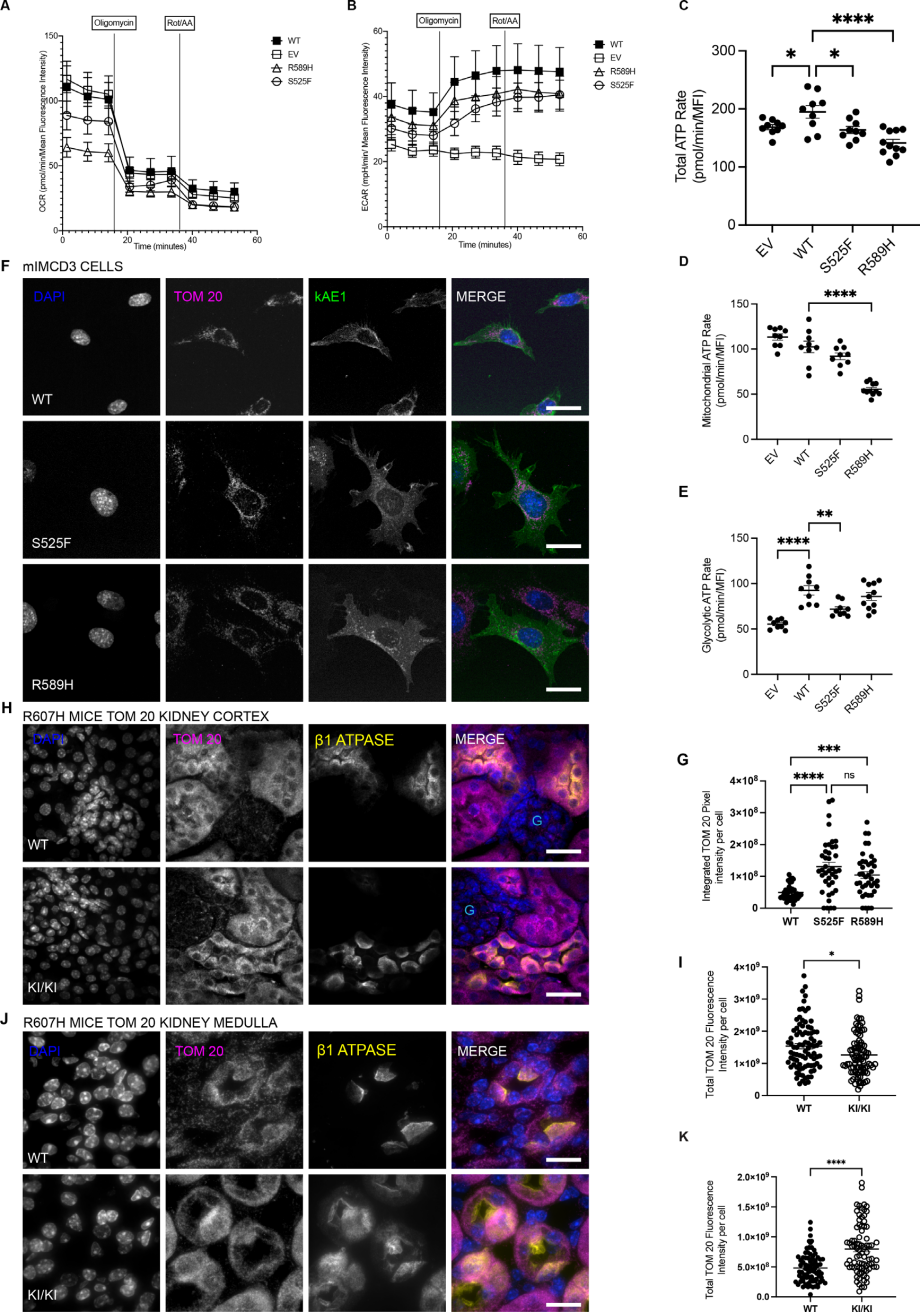
dRTA kAE1 mutant cells have lower ATP production rate and abnormal mitochondrial content. Oxygen consumption rate (OCR) (**A**) and extra cellular acidification rate (ECAR) (**B**) of empty vector (EV) transfected cells, kAE1 WT, S525F, or R589H mIMCD3 cells analyzed in a Seahorse XFe96 Extracellular Flux Analyzer with the ATP Rate Assay Test Kit. All cell lines, including EV-transfected cells, were incubated with doxycycline to eliminate a potential effect of doxycycline on measurements. (**C**) Graphical representation of the combination of ATP production rates from mitochondrial respiration (mitoATP) and glycolysis (glycoATP) of kAE1 WT, S525F, and R589H mIMCD3 cells measured in real-time following sequential injections of oligomycin and Rotenone + Antimycin-A. Error bars correspond to mean ± SEM, n=minimum. *p<0.05, ****p<0.0001 using one-way ANOVA followed by Tukey’s post hoc test. Graphical representations of mitochondrial respiration (**D**) and glycolytic ATP production (**E**) in kAE1 WT, S525F, and R589H mIMCD3 cells. Error bars correspond to mean ± SEM, n=minimum 8. **p<0.01, ****p<0.0001 using one-way ANOVA followed by Tukey’s post hoc test. (**F**) Immunofluorescence staining of TOM20 and kAE1 in kAE1 WT, S525F, and R589H mIMCD3 cells. Blue = nuclei, magenta = TOM20, green = kAE1. Scale bar = 8 µm. (**G**) Graphical representation of total TOM20 fluorescence intensity per cell expressing kAE1. Error bars correspond to mean ± SEM, n=minimum 40. ***p<0.001, ****p<0.0001 using one-way ANOVA followed by Tukey’s post hoc test. Immunofluorescence images of TOM20 and ß1 ATPase in kidney cortex (**H**) or medulla (**J**) of kAE1 R607H WT and KI mice exposed to a salt-depleted diet with an acid challenge (Mungara et al., 2024). Blue = nuclei, magenta = TOM20 (mitochondria), yellow = ß1 ATPase, light blue ‘G’ indicates the location of a glomerulus. Scale bar = 8 μm. Graphical representation of the total TOM20 fluorescence intensity in ß1 ATPase-positive cells in the cortex (**I**) or medulla (**K**) of the kidney. Error bars correspond to mean ± SEM, n=90. *p<0.05, ****p<0.0001 using Student’s *t*-test.

Lysosomal degradation is highly dependent on a low luminal pH generated in part by the vacuolar-type H^+^-ATPase (Ratto et al., 2022) whose activity depends on ATP hydrolysis. We therefore analyzed the ATP production rate in mIMCD3 cells, specifically glycolysis and oxidative phosphorylation. We measured the oxygen consumption rate (OCR) (Figure 5A) and extracellular acidification rate (ECAR) (Figure 5B) in empty vector-transfected, kAE1 WT, or mutant cells. Both kAE1 S525F and R589H mutant cells had a lower ATP production rate compared to WT (Figure 5C). More specifically, the R589H mutant cells had a lower mitochondrial ATP production rate (Figure 5D), whereas the kAE1 S525F mutant cells exhibited a lower glycolytic ATP production rate (Figure 5E). With the mitochondria being the major ATP-producing organelles (Jonckheere et al., 2012), we assessed mitochondrial content by immunostaining of translocase of the outer membrane 20 (TOM20) both in vitro and in vivo. Both kAE1 S525F and R589H mutant cells have higher mitochondrial content compared to WT as determined by total overall intensity of TOM20-positive puncta (Figure 5F and G). In line with this result, although a decreased fluorescence intensity was observed in the cortex, there was a significantly higher TOM20 fluorescence intensity in medullary kidneys of homozygous R607H KI mutant mice compared to WT littermates (Figure 5H–K).

## Discussion

In this study, we characterized three newly identified dRTA-causing kAE1 variations. Combining in vivo and in vitro studies, we demonstrated that reduced transport activity of the kAE1 mutants correlated with increased cytosolic pH, reduced ATP synthesis, attenuated downstream autophagic pathways, and lysosomal dysfunction, pertaining to the fusion of autophagosomes and lysosomes and/or lysosomal degradative activity (see summary in Tables 1 and 2).

**Table 1.** Summary of findings in dRTA kAE1 variant expressing cells compared to WT.

dRTA variant	Transport activity	Intracellular pH	LC3B accumulation	Autophagy flux	Cellular lysosome size	Cellular lysosome number	Cellular mitochondrial abundance	Rescued autophagy
*R295H*	Unchanged	Unchanged	Unchanged	N/A	N/A	N/A	N/A	N/A
*S525F*	Reduced	Increased	Increased	Blocked downstream	Unchanged	Increased	Increased	Yes
*R589H*	Slightly Reduced	Increased	Increased	Blocked downstream	Increased	Unchanged	Increased	Yes

N/A, not applicable.

**Table 2.** Summary of findings in intercalated cells from dRTA R607H knock-in (KI) relative to WT mice.

Genotype	LC3B accumulation	Intercalated cell lysosome size	Intercalated cell lysosome number	Intercalated cell mitochondrial abundance
*R607H KI/KI*	Increased total LC3B	Increased	Increased	Increased

In line with previous observations in mIMCD3 cells (Mumtaz et al., 2017), the kAE1 R295H, Y413H, and S525F mutants were properly localized to the basolateral membrane after polarization. When expressed in Madin-Darby canine kidney (MDCK) cells, other dRTA-causing kAE1 mutants such as dRTA R602H, G701D, V488M, deltaV850 variants exhibited a plasma membrane trafficking defect (Sawasdee et al., 2006; Yang et al., 2023; Deejai et al., 2022). In contrast, the kAE1 R589H mutant was correctly targeted to the plasma membrane (Mumtaz et al., 2017). Functionally, cells expressing the Y413H and S525F mutants exhibit about 60% reduction in chloride/bicarbonate exchange activity compared to kAE1 WT, similar to the previously characterized recessive G701D mutant but unlike the R295H mutant (Chu et al., 2013). Therefore, the mechanism causing dRTA remains unclear in the case of the newly identified recessive R295H variant. Additionally, the kAE1 Y413H variant exhibited a shorter half-life than the WT counterpart, likely explaining dRTA, and thus was not further investigated. These findings add to the growing list of *SLC4A1* gene variations causing dRTA.

We next investigated the roots of the altered autophagy briefly reported in R607H KI mice (Mumtaz et al., 2017) using mIMCD3 cells and whole kidney lysates. In the kidneys of the KI mice, a decrease in the number of A-IC, accumulation of p62 and ubiquitinylated proteins, and enlarged remaining A-ICs suggested altered autophagy processes in dRTA mutant cells and in homozygous R607H KI mice (Mumtaz et al., 2017; Chu et al., 2013). During the autophagy process, LC3B I (a marker for autophagosomes) is converted to lipidated LC3B II (Dhingra et al., 2018) and p62 aggregates to facilitate the degradation of ubiquitinated proteins within the autophagosome complex (Huang et al., 2023). In mIMCD3 cells expressing dRTA kAE1 S525F and R589H variants, LC3B lipidation was increased compared to WT, an increase that persisted with both Baf A1 treatment and starvation. Although opposite effects were expected under inhibition or induction of autophagy, such similar effect has been previously described. In the proximal tubule of obese mice, LC3B accumulation indicating a stagnated autophagy flux was observed with both chloroquine treatment and 24-hour starvation (Yamamoto et al., 2017). Similarly, in NRK-52E cells, a disruption of the autophagy machinery in high cadmium-stressed cells resulted in LC3B II accumulation under either Baf A1 or rapamycin (an autophagy inducer) treatment (Lee et al., 2017). Although LC3B II is elevated during both increased autophagy flux and disrupted autophagy, we did not observe significant accumulation of p62 in mIMCD3 cells expressing dRTA mutants. p62 is specifically a marker of autophagy-mediated protein clearance (Brown et al., 2021; Mizushima et al., 2002; Klionsky et al., 2012). Therefore, p62 accumulation in R607H KI mouse kidney sections strongly suggests a compromised autophagy-mediated clearance, while the increased LC3B lipidation without significant changes in p62 in mIMCD3 cells points towards either an increased autophagic flux and/or a disrupted autophagy.

To obtain a clearer picture of the precise autophagic pathway altered in the dRTA mutants, we probed further into the different stages of autophagy and autophagy flux. We noted an accumulation of autophagosomes and autolysosomes in the S525F and R589H mutant cells. This was recapitulated in the R607H KI mice which showed significantly more and larger LAMP1-positive vesicles in both kidney cortex and medulla, suggesting a blockage in late steps of autophagy flux in both dRTA mutant cells and KI mice. Such blockage has been implicated in the pathophysiology of several diseases. In lysosomal storage disease, lysosome accumulation in proximal tubule cells is a key component in the pathways mediating epithelial dysfunction (Festa et al., 2018). In this study, restoring autophagy flux attenuated disease progression. Another study in SK-N-SH, RT4-D6P2T, and HeLa cells implicated autophagosome and lysosome accumulation in cellular toxicity associated with neurodegenerative diseases (Button et al., 2017). In agreement with altered lysosomal function, we also noted a greater abundance and size of active cathepsin B lysosomal protease vesicles in mIMCD3 cells. Increased cathepsin B activity affects lysosomal biogenesis, autophagy initiation, and cellular homeostasis (Liu et al., 2024). In the renal context, cathepsin B knockout mice demonstrated a higher resistance and quicker recovery from glomerular damage (Höhne et al., 2018), suggesting that cathepsin B accumulation may be detrimental to the cells. Increased cathepsin B abundance in the dRTA mutant cells also correlates with the accumulation of lysosomes. Overall, these findings suggest that the pathogenesis of dRTA in our in vitro and in vivo models involves an inhibition of autophagy flux at the downstream steps involving autophagosome-lysosome fusion and lysosomal protein clearance (Ballabio and Gieselmann, 2009; Eskelinen, 2006; Mizushima, 2018; de Araujo et al., 2020).

The question remained as to how these dRTA variants altered autophagy. The *SLC4A1-3* gene family that includes AE1 are regulators of intracellular pH in different cell types (Thornell and Bevensee, 2015; Zhang et al., 2023; Romero et al., 2013). The reduced anion exchange activity in mIMCD3 cells expressing the R589H and S525F variants expectedly correlated with an increased pHi compared to WT counterparts. Given that pHi variations can impact autophagy (Berezhnov et al., 2016; Korolchuk et al., 2011; Heuser, 1989; Xu et al., 2011; Ratto et al., 2022), we wondered whether this was the case for cells expressing kAE1 dRTA mutants. We observed that an acidic pHi in mutant cells restored autophagy levels similar to WT. Previous studies reported more perinuclear localization of lysosomes and autophagosome-lysosome fusion in cells with an increased pHi (Korolchuk et al., 2011; Heuser, 1989). Although not examined in our study, an increase in intracellular pH due to starvation decreased the levels of lysosomal kinesin superfamily member KIF2 and ADP-ribosylation factor-like 8B (ARL8), which are responsible for redistributing lysosomes to the cell periphery. This reduction subsequently inhibited mTORC1, resulting in increased autophagosome synthesis and autophagosome-lysosome fusion (Korolchuk et al., 2011).

While an alkaline cytosolic pH partially explains the impairment in autophagy flux, a close relationship also exists between intracellular pH and cellular energy dynamics and metabolic stress, all of which are key regulators of autophagy (Mizushima and Komatsu, 2011). Therefore, it was plausible that the kAE1 variant-induced autophagy dysregulation occurs through a signaling pathway akin to that of energy deprivation-induced autophagy. This hypothesis is supported by our findings of reduced ATP production rate in dRTA kAE1 S525F and R589H mutant cells compared to kAE1 WT cells. We also found that both kAE1 S525F and R589H mutant cells have higher mitochondrial content compared to kAE1 WT cells. The increased mitochondrial content coupled with low ATP points towards improper mitochondrial function in dRTA variant cells. Low ATP levels as seen in dRTA mutant cells may impair autophagy, as was shown in human RPE cells (Schütt et al., 2012). ATP reduction in RPE cells led to complex mitochondrial changes such as structural disorganization, enzyme activity decline, and oxidative damage to mitochondrial components and DNA (Schütt et al., 2012). Similarly, in pancreatic islet cells, an alkaline pHi led to increased uptake of phosphate by mitochondria, accelerating the production of superoxide, promoting mitochondrial permeability transition, and inducing translational attenuation due to endoplasmic reticulum stress, ultimately impairing insulin secretion (Nguyen et al., 2016). Overall, our data support that expression of the kAE1 variants increases pHi, which alters mitochondrial function and leads to reduced cellular energy levels that eventually attenuate energy-dependent autophagic pathways including autophagosome-lysosome fusion and lysosomal protein clearance.

In light of these observations, we postulated that correcting the alkaline pHi of dRTA mutant-expressing mIMCD3 cells will alleviate this blockage in autophagy flux. We observed that a chemically engineered pHi of 6.9 (Lyons et al., 1992) reduced LC3B II accumulation and LAMP1 abundance in mIMCD3 mutant cells to expression levels similar to that of WT cells at baseline. This suggests that a chemically reduced pHi facilitated protein clearance in the two dRTA mutant cells (Berezhnov et al., 2016; Korolchuk et al., 2011) as noted in other studies. In one such study, treatment of SH-SY5Y cells with FCCP and nigericin also acidified intracellular pH and triggered autophagy and mitophagy (Berezhnov et al., 2016). Similarly, acid loading in proximal tubular cells under chronic metabolic acidosis showed increased autophagic flux and mitophagy (Namba et al., 2014). These findings are in line with our results and establish a link between altered autophagy flux and the alkaline pHi of dRTA variant cells. Overall, our study provides one pathway (altered pHi) by which dRTA may arise. However, different variants induce different degrees of functional defects as seen in Figure 1F & G. The kAE1 R295H, the only reported amino acid substitution in the amino-terminal cytosol causing dRTA, does not affect the transporter’s function or pHi. Therefore, this variant may cause dRTA via a different pathway, for example, defective protein–protein interactions, than transport-defective S525F or partially inactive R589H variants.

In conclusion, our study established a strong relationship between the expression of defective kAE1 proteins, reduced mitochondrial activity, decreased autophagy, and impaired protein degradative flux. Whether this abnormal degradative pathway explains the premature loss of A-IC will need to be elucidated in further studies.

## Materials and methods

### Antibodies and chemicals

Mouse anti-HA antibody (hemagglutinin, Biolegend, formerly Covance), mouse anti-β-actin antibody (Sigma Aldrich or anti-β-actin HRP clone 2F1-1 Biolegend cat#643807), mouse anti-LC3B antibody (Cell Signaling), mouse anti-IVF 12s antibody (Developmental Studies Hybridoma Bank), rat anti-ATP6V1B1 antibody (BiCell #20901), rabbit anti-mTOR antibody and phospho mTOR antibody (Cell Signaling), rabbit anti-4E-BP1 antibody and phospho 4E-BP1 (Ser65) antibody (Cell Signaling), mouse anti-p62 antibody (Abcam), mouse anti-p53 antibody (Cell Signaling), rabbit anti-Cleaved caspase 3 antibody (Cell Signaling), mouse monoclonal anti Na^+^/K^+^-ATPase H-3 antibody (Santa Cruz Biotechnology, Dallas, TX), goat anti-mouse antibody horseradish peroxidase conjugated (HRP) (Cell Signaling Technology), Cy3-conjugated donkey anti-mouse antibody, anti-rabbit and anti-goat antibodies (Jackson Immunoresearch), X-tremeGENE.

### Newly identified *SLC4A1* variations from dRTA patients

The patient carrying the R295H mutation was a boy carrying the variation in the homozygous state, whose genetic diagnosis was made at the age of 5, following growth retardation of –2 SD for both weight and height, with bicarbonate at 18 mmol/L, potassium at 2.8 mmol/L, chloridemia at 94 mmol/L, calcemia at 2.55 mmol/L, and a urinary pH of 7.5. He also had a history of a pyeloureteral junction syndrome that was surgically managed at the age of 3. The R295H dRTA variation is a nonsense homozygous substitution characterized by a replacement of guanine (G) on position 884 by adenine (A) in the coding sequence. It has an allelic frequency of 0.14% in the European population.

For the Y413H variant, the patient was a female diagnosed at 1 month old, with a urinary pH of 7.5, evidence of nephrocalcinosis, and failure to thrive. The S525F variant has been previously reported (Bertocchio et al., 2020), but in brief, the patient was a 13-year-old female with plasma pH of 7.25, plasma bicarbonate at 15.3 mmol/L who also presented with polyurethral junction syndrome, nephrocalcinosis, and nephrolithiasis since childhood. The Y413H and S525F dRTA variations are nonsense heterozygous substitutions characterized by a replacement of thymine (T) at position 1237 by cytosine (C) and a replacement of cytosine (C) at position 1574 by thymine (T), respectively. The R589H dRTA variation has been previously described (Mumtaz et al., 2017). No follow-up data were available for all patients.

### Mice

Transgenic mice carrying the R607H knockin (KI) mutation (murine equivalent to human R589H mutation) were previously described (Mumtaz et al., 2017). Homozygous mice used throughout the study display incomplete dRTA with alkaline urine without metabolic acidosis at baseline as previously reported (Mumtaz et al., 2017). Homozygous mice or wild-type (WT) littermates were fed a standard rodent chow (Picolab Rodent Diet 20 # 5053, LabDiet, ST. Louis, MO, USA) or for Figure 5H-K, a salt-depleted diet with acid challenge as previously reported (Mungara et al., 2024) with adequate and constant water supply, and maintained on a 12hour light and dark cycle throughout their lifespan.

### Cell lines, transfections, and viral infection

Mouse inner medullary collecting duct (mIMCD) cells (ATCC# CRL2123) were used for preparing kAE1 wild type and mutant cell lines. The pLVX TRE3G kAE1 construct was generated from the shuttling of human kAE1 cDNA with an external hemagglutinin (HA) epitope in position 557 (on eAE1) into pLVX-TRE3G plasmid (Clontech) (Lashhab et al., 2019). This construct encodes a protein described as kAE1 throughout this paper. The kAE1 S525F and R589H mutants were generated with Q5 site-directed mutagenesis. All plasmids were introduced into the mIMCDs using a viral single-shot packaging kit (Clontech).

### Mouse intercalated cell isolation and tissue homogenate preparation

Kidney tissue homogenates were prepared as previously described (Mungara et al., 2025). Briefly, after decapsulation, freshly dissected kidneys were homogenized in cold lysis buffer (0.3 M sucrose, 25 mM imidazole, 1 mM EDTA, 8.5 µM leupeptin, 1 mM PMSF), and vortexed over 1 hour every 15 min. The homogenates were then centrifuged at 14,000 rpm for 15 minutes at 4°C prior to measurement of protein concentration by Bicinchoninic Acid Protein Assay. Primary intercalated cells were prepared from homozygous kAE1 R607H transgenic mice. After cardiac perfusion with PBS, heparin, and collagenase B (Sigma Aldrich), kidneys were homogenized by MACS dissociation and intercalated cells enriched using CD 117 magnetic sorting (Miltenyi Biotec) as previously described (Saxena et al., 2021). During the selection, intercalated cells were kept in MACS buffer (PBS, 2 mM EDTA, and 0.5% FBS). Cells were lysed with RIPA lysis buffer (2 mM EDTA, 2% deoxycholate, 0.3 M NaCl, 20 mM Tris/HCl pH 7.5, 2% Triton X-100, 0.2% SDS, pH 7.4), supplemented with complete EDTA-free protease inhibitors, and PhoSTOP phosphatase inhibitor (Roche), PMSF, pepstatin, leupeptin, and aprotinin. An aliquot was saved for bicinchoninic acid assay to determine protein concentration, and remaining lysates were kept in Laemmli buffer at –20°C for immunoblotting.

### Bicarbonate transport assay

This assay has been previously described (Sterling and Casey, 1999). Briefly, confluent kAE1 WT-HA or mutant mIMCDs cells grown on coverslips were incubated with 1 μg/mL doxycycline (Sigma-Aldrich) for 18–24 hours at 37°C to induce kAE1 expression. They were then incubated with 2',7'-Bis-(2-Carboxyethyl)–5-(and-6)-Carboxyfluorescein, Acetoxymethyl Ester (BCECF–AM, Thermo Scientific), a fluorophore which excites at 440 and 490 nm and emits 510 nm wavelength for 30 minutes at 37°C. Using a fluorometer from Photon Technologies International (PTI) (London, Ontario, Canada), coverslips were perfused with NaCl-based Ringer’s buffer (5 mM glucose, 5 mM potassium gluconate, 1 mM calcium gluconate, 1 mM magnesium sulfate, 10 mM HEPES, 2.5 mM sodium dihydrogen phosphate, 25 mM sodium bicarbonate, 140 mM sodium chloride) for 5–10 minutes. Once stable, initial fluorescence (corresponding to steady-state pHi) was recorded for the first 30 seconds before switching to chloride-free containing sodium gluconate-based Ringer’s buffer of same osmolality. BCECF fluorescence was calibrated by perfusing cells with different pH buffers (6.5, 7, 7.5) in the presence of 10 mM nigericin. The Ringer’s buffers were continuously bubbled with an air:CO_2_ mixture (19:1), providing 5% CO_2_. Transport rates of the cells were determined by linear regression of the initial fluorescence variations (over the first 60 seconds), normalized to pH calibration measurements. All measurements were done using PTI FelixGX software.

### Cell treatments and immunoblotting

For autophagy experiments, kAE1 WT or mutant cells were seeded to 70% confluency on 10 cm culture plates and treated with 1 μg/mL doxycycline (Sigma-Aldrich) for 48 hours. Cells were then either treated with 400 nM bafilomycin A1 for 4 hours to inhibit autophagy or with Hanks balanced salt solution (HBSS, Gibco) to starve cells and induce autophagy or given no treatment. To chemically modify pHi of cells, 90–100% confluent cells were treated with 1 µg/mL of doxycycline (Sigma-Aldrich) overnight. Cells were then incubated in Ringer’s buffer with pH 6.6, supplemented with 0.03 μM nigericin with a final potassium concentration of 168 mM for 2 hours at 37°C. Steady-state cells were incubated in normal pH media without nigericin prior to lysis. Under treated conditions, pHi was similar to pHe (Figure 3—figure supplement 1). Cells were lysed with RIPA lysis buffer (1% deoxycholate, 1 mM EDTA, 0.15 M NaCl, 0.1% SDS, 10 mM TRIS/HCl [pH 7.5], 1% Triton X-100) with phosphatase inhibitors (cat. no. 04906837001; Roche PhosSTOP) and protease inhibitors (cat. no. 04693159001; Roche Complete Tablets, Mini EDTA-free) and stored at −20°C with or without 2x Laemmli buffer. The aliquot without Laemmli buffer was used for a bicinchoninic acid (BCA) assay to determine protein concentration. Following the BCA, 10–30 mg of total protein was loaded on SDS-PAGE gels. Proteins were transferred to PVDF membranes and antibodies listed above were used for detecting the proteins of interest. Primary antibodies were diluted in 1% milk and incubated on membranes overnight at 4°C followed by secondary antibodies linked with horseradish peroxidase (HRP) for 1 hour at room temperature. Protein detection was done with the Enhanced Chemiluminescence reagent (ECL Prime, Invitrogen), and a BioRad Imager. The ImageLab software (BioRad) was utilized for the quantification of relative band intensities.

### Cell surface biotinylation assay

mIMCD3 cells stably expressing kAE1 WT, S525F, or R589H were seeded to 70–80% confluency. The cells were incubated with sulfo-N-hydroxysuccinimide-SS-biotin (Thermo cat.# 21331) (1.5 mg/mL in ice-cold PBS) for an hour at 4°C, quenched with 100 mM glycine and lysed with RIPA lysis buffer, supplemented with complete EDTA-free protease inhibitors, and PhoSTOP phosphatase inhibitor (Roche), PMSF, pepstatin, leupeptin, and aprotinin. Total protein levels were measured by BCA, and an aliquot was saved as ‘Total’ fraction. 450 mg of each lysate was subsequently incubated with 100 μL streptavidin slurry beads for 1 hour on a rocker at 4°C. Following centrifugation, the supernatant was collected, and an aliquot kept as the ‘unbiotinylated’ fraction. After six washes, the beads were resuspended in 50 μL of 2X Laemmli buffer and incubated at room temperature for 30 minutes. The eluted biotinylated proteins were subsequently collected by centrifugation (‘Biotinylated’ fraction). The biotinylated fraction (45 μL) was loaded on SDS-PAGE gel for immunoblot analysis along with 3 μg of the Total fraction and a matched volume of unbound fraction per well. In addition to anti-HA antibody, the blots were probed for actin to ensure cell membrane integrity was intact during the biotinylation procedure, and for Na^+^/K^+^-ATPase as cell surface control.

### Magic Red assay

80% confluent kAE1 WT or mutant mIMCD3 cells were treated with 1 μg/mL doxycycline (Sigma-Aldrich) for 48 hours to induce kAE1 expression. Different treatments, including a 4-hour incubation with 400 nM bafA1 to inhibit autophagy, a 2-hour starvation in HBSS to induce autophagy, or no treatment (steady state), were applied. Cells were then incubated with 1% Magic Red reagent (ImmunoChemistry Technologies) in DMEM-F12 medium at 37°C in the dark for 30–60 minutes. Cells were then fixed with 4% PFA, quenched with 100 mM glycine, permeabilized with 0.2% Triton X-100, blocked with 1% BSA, and incubated with mouse anti-HA primary antibody and donkey anti-mouse Alexa Fluor 488 conjugated secondary antibody for 30 minutes each. Cells were then incubated with 4′,6-diamidino-2-phenylindole (DAPI) for 5 minutes before mounting using DAKO Mounting Medium (Agilent Technologies). A WaveFX confocal microscope was used to image the slides, and the images were analyzed blindly using the Fiji software.

### Autophagy flux assay

kAE1 WT-HA or mutant mIMCD3 cells seeded to 70% confluency in a 6-well plate on coverslips were transiently transfected with the eGFP-RFP-LC3 cDNA construct (kind gift from Dr. Goping, Department of Biochemistry, University of Alberta) using the X-tremeGENE HP DNA transfection reagent (Roche). 4 hours after transfection, the cells were incubated with 1 μg/mL doxycycline for 48 hours at 37°C to induce kAE1 expression. Following this incubation, cells were incubated with blocking medium, mouse anti-HA primary antibody (1:200), and donkey anti-mouse Alexa Fluor 649 (Jackson ImmunoResearch). Hoechst stain (ImmunoChemistry Technologies) was used to stain cellular nuclei. A WaveFX confocal microscope together with the Velocity and Fiji software was used to capture and analyze images.

### Assessment of mitochondrial content

kAE1 WT-HA or mutant mIMCD3 cells seeded to 50% confluency in a 6-well plate on coverslips were incubated with 1 μg/mL doxycycline for 48 hours at 37°C and overnight in complete medium with no antibiotics. Cells were then fixed with 4% PFA, quenched with 100 mM glycine, permeabilized with 0.2% Triton X-100, blocked with 1% BSA, and incubated with mouse anti-HA and rabbit anti-TOM20 primary antibodies and then with donkey anti-mouse Alexa Fluor 488 and anti-rabbit Cy3 conjugated secondary antibodies. Cells were then incubated with DAPI for 5 minutes before mounting using DAKO Mounting Medium (Agilent Technologies). A WaveFX confocal microscope was used to image the slides, and the images were analyzed blindly using the Fiji software.

### Metabolic flux analysis

kAE1 WT-HA or mutant mIMCD3 cells were treated with 1 μg/mL doxycycline for 48 hours followed by an overnight incubation in complete media with no antibiotics and cells seeded at 2 × 10^4^ cells per well in Seahorse XFe 96-well plates overnight to form a uniform monolayer. On the day of assay, culture medium was replaced with XF DMEM Medium pH 7.4 (103575-100, Agilent Technologies) with glucose (10 mM), pyruvate (1 mM), and L-glutamine (2 mM) and incubated in a non-CO_2_, 37°C incubator for 1 hour prior to their placement into the XFe96 Analyzer. Using the ATP production rate assay kit (#103592-100, Agilent Technologies) and XF cell Mito Stress Test kit (#103015-100, Agilent Technologies), metabolic indices were obtained from the Seahorse XFe96 Analyzer following manufacturer’s procedures previously described (Sawasdee et al., 2010). The total ATP rate is the sum of ATP production rate from both glycolysis and oxidative phosphorylation. Glycolysis releases protons in a 1:1 ratio with ATP; hence, the glycolytic ATP rate is calculated from the glycolytic proton efflux rate (glycoPER). GlycoPER is determined by subtracting respiration-linked proton efflux from total proton efflux by inhibiting complex I and III. The empty vector transfected cells provided a control for a potential effect of doxycycline on measurements. Oxygen consumption rate (OCR) and extracellular acidification rate (ECAR) were measured at various time points at basal state followed by injections of oligomycin (1.5 μM) and Rotenone + Antimycin A (0.5 μM).

### Tissue preparation and immunostaining of kidney sections

Kidneys collected after perfusion were stored in 4% PFA overnight at 4°C. The PFA solution was switched to 15% sucrose for 2 hours and then transferred to 30% sucrose solution overnight at 4°C. Thereafter, kidneys were fixed in O.C.T (Tissue-Tek) and snap frozen in liquid nitrogen. These tissues were stored at –80°C until cryo-sectioning. Ten (10) micron tissue sections were fixed on a charged glass slide (Thermo Fisher) and used immediately or stored at –80°C until immunostaining. For immunostaining, the slices were first air-dried for 20 minutes, washed with PBS for 5 minutes, and fixed with 4% PFA for 20 minutes at 4°C. The sections were quenched with 100 mM glycine for 15 minutes, permeabilized, and blocked with 5% or 10% serum in 0.2% Triton in PBS for 1 hour at room temperature. Slices were incubated in primary antibody diluted in 5% or 10% serum overnight at 4°C followed by secondary antibody diluted in 5% or 10% serum for 1 hour at room temperature. Slices were washed with 0.1% Tween 20 in PBS and incubated with DAPI for 5 minutes at room temperature, mounted with DAKO mounting medium, and sealed. Slides were air-dried and then stored at –20°C. Note that kidney sections analyzed in Figure 5H–K were obtained from mice fed a ‘salt-depletion with acid load’ diet consisting of a low sodium and chloride diet for 8 days, complemented with 0.28 M NH_4_Cl with 0.5% sucrose in drinking water for six additional days as previously described (Escobar et al., 2016). This diet triggered a metabolic acidosis, significantly lower plasma bicarbonate with a more alkaline urine in the homozygous KI mice compared to WT littermates.

### Confocal imaging and image analysis

Immunofluorescent imaging was done with a WaveFX confocal microscope (Quorum Technologies, Guelph, Ontario, Canada) powered by a Volocity software (Quorum Technologies). Images were taken with ×40 oil immersion objective with z-stacks at 0.5 µm intervals. Quantitative image analysis was performed using the Volocity analysis software or by open-source cell image analysis software CellProfiler (Stirling et al., 2021) and Fiji (Schindelin et al., 2012).

### Image analysis

**Cell profiler** was used to analyze TOM20 staining in mIMCD3 cells. After converting the three-channel RGB images to grayscale using the split method, we manually outlined each cell in the channel corresponding to kAE1 staining and inputted it back into the pipeline. The objects were then converted to a binary image and the TOM20 channel was used as the input channel to measure intensity of TOM20 staining in the manually outlined objects. The raw data, including the sum of TOM20 pixel intensity per cell, were then analyzed using GraphPad Prism software. A minimum of 30 cells were analyzed.

**Fiji** software was used to analyze images from Magic Red staining and autophagy flux experiment. After each multichannel image was opened and merged in Fiji, the ‘multi point’ tool was used to label all regions of interest. Freehand selection tool was used to manually draw the outline of all selected regions of interest, followed by the ‘Analyze’ command to extract number and size of puncta in pixels. Pixel values were then converted to microns and data analysis was completed using GraphPad Prism. A minimum of 30 cells was analyzed.

**Volocity** software was used for analysis of kidney sections. From B1 H^+^-ATPase-positive single cells cropped from confocal images of medullary or cortical mouse kidney sections, the channel of interest was used to find objects using the ‘Find objects’ command. Refinement within the selection was made based on object size. The minimum object size thresholds were 0.016 μm^2^ for LAMP1 puncta and 0.02 μm^2^ for TOM20 staining. This was then labeled as ‘population one’. From population one, touching objects were separated using a size guide of 0.02 μm^2^ for LAMP1 puncta only with the ‘Separate touching objects’ function. For TOM20 staining, all RFP-positive stain within individual cells was characterized under one mask without separating touching objects. The minimum object size was set to 0.02 mm^2^ and everything less than that was considered background staining. The sum of TOM20 fluorescence intensity per cell was collected and analyzed using GraphPad Prism. For LAMP1 images, an exclusion criterion removing objects lesser than or equal to 0.02 mm^2^ was used to remove small background objects. Once the regions of interest in the image were properly outlined, data, including number of puncta, intensity of puncta, area/ volume of puncta, among other measurements, were exported and data analyzed with GraphPad Prism. A minimum of 60 cells was analyzed.

### Statistical analysis

All the experiments were independently repeated a minimum of three times. Experimental results were analyzed using the GraphPad Prism software and are summarized as mean ± SEM. All statistical comparisons were made using unpaired Student’s *t*-test or one/two-way ANOVA followed by a post hoc test as indicated in figure legends. A p-value <0.05 was considered statistically significant. All datasets were assessed for normality, and outliers identified by Prism were excluded.

## Data Availability

Source Data (including Western Blot source data) are available at: https://doi.org/10.5061/dryad.2bvq83c4f. The following dataset was generated: EssumanG RizviM AlmomaniE UllahSAKM HasibSMA ChelangarimiyandoabF MungaraP SchmittMJ HureauxM Vargas-PoussouR TouretN CordatE Dryad Digital Repository10.5061/dryad.2bvq83c4f2026Data: SLC4A1 mutations that cause distal renal tubular acidosis alter cytoplasmic pH and cellular autophagyPMC1295988241778592

## Additional files

MDAR checklist

## References

[bib1] Ballabio A, Gieselmann V (2009). Lysosomal disorders: from storage to cellular damage. Biochimica et Biophysica Acta.

[bib2] Berezhnov AV, Soutar MPM, Fedotova EI, Frolova MS, Plun-Favreau H, Zinchenko VP, Abramov AY (2016). Intracellular pH modulates autophagy and mitophagy. The Journal of Biological Chemistry.

[bib3] Bertocchio J-P, Genetet S, Da Costa L, Walsh SB, Knebelmann B, Galimand J, Bessenay L, Guitton C, De Lafaille R, Vargas-Poussou R, Eladari D, Mouro-Chanteloup I (2020). Red blood cell AE1/Band 3 transports in dominant distal renal tubular acidosis patients. Kidney International Reports.

[bib4] Brown CN, Atwood D, Pokhrel D, Holditch SJ, Altmann C, Skrypnyk NI, Bourne J, Klawitter J, Blaine J, Faubel S, Thorburn A, Edelstein CL (2021). Surgical procedures suppress autophagic flux in the kidney. Cell Death & Disease.

[bib5] Button RW, Roberts SL, Willis TL, Hanemann CO, Luo S (2017). Accumulation of autophagosomes confers cytotoxicity. The Journal of Biological Chemistry.

[bib6] Chang YH, Shaw CF, Jian SH, Hsieh KH, Chiou YH, Lu PJ (2009). Compound mutations in human anion exchanger 1 are associated with complete distal renal tubular acidosis and hereditary spherocytosis. Kidney International.

[bib7] Cheung JC, Cordat E, Reithmeier RAF (2005). Trafficking defects of the Southeast Asian ovalocytosis deletion mutant of anion exchanger 1 membrane proteins. The Biochemical Journal.

[bib8] Chu CYS, King JC, Berrini M, Alexander RT, Cordat E (2013). Functional rescue of a kidney anion exchanger 1 trafficking mutant in renal epithelial cells. PLOS ONE.

[bib9] Cordat E (2006). Unraveling trafficking of the kidney anion exchanger 1 in polarized MDCK epithelial cells. Biochemistry and Cell Biology = Biochimie et Biologie Cellulaire.

[bib10] Cordat E, Reithmeier RAF (2014). Structure, function, and trafficking of SLC4 and SLC26 anion transporters. Current Topics in Membranes.

[bib11] de Araujo MEG, Liebscher G, Hess MW, Huber LA (2020). Lysosomal size matters. Traffic.

[bib12] Deejai N, Sawasdee N, Nettuwakul C, Wanachiwanawin W, Sritippayawan S, Yenchitsomanus PT, Rungroj N (2022). Impaired trafficking and instability of mutant kidney anion exchanger 1 proteins associated with autosomal recessive distal renal tubular acidosis. BMC Medical Genomics.

[bib13] Dhingra A, Alexander D, Reyes-Reveles J, Sharp R, Boesze-Battaglia K (2018). Microtubule-associated protein 1 light chain 3 (LC3) isoforms in RPE and retina. Advances in Experimental Medicine and Biology.

[bib14] Duangtum N, Junking M, Sawasdee N, Cheunsuchon B, Limjindaporn T, Yenchitsomanus P (2011). Human kidney anion exchanger 1 interacts with kinesin family member 3B (KIF3B). Biochemical and Biophysical Research Communications.

[bib15] Enerbäck S, Nilsson D, Edwards N, Heglind M, Alkanderi S, Ashton E, Deeb A, Kokash FEB, Bakhsh ARA, Van’t Hoff W, Walsh SB, D’Arco F, Daryadel A, Bourgeois S, Wagner CA, Kleta R, Bockenhauer D, Sayer JA (2018). Acidosis and deafness in patients with recessive mutations in FOXI1. Journal of the American Society of Nephrology.

[bib16] Escobar LI, Simian C, Treard C, Hayek D, Salvador C, Guerra N, Matos M, Medeiros M, Enciso S, Camargo MD, Vargas-Poussou R (2016). Mutations in ATP6V1B1 and ATP6V0A4 genes cause recessive distal renal tubular acidosis in Mexican families. Molecular Genetics & Genomic Medicine.

[bib17] Eskelinen EL (2006). Roles of LAMP-1 and LAMP-2 in lysosome biogenesis and autophagy. Molecular Aspects of Medicine.

[bib18] Festa BP, Chen Z, Berquez M, Debaix H, Tokonami N, Prange JA, van de Hoek G, Alessio C, Raimondi A, Nevo N, Giles RH, Devuyst O, Luciani A (2018). Impaired autophagy bridges lysosomal storage disease and epithelial dysfunction in the kidney. Nature Communications.

[bib19] Giglio S, Montini G, Trepiccione F, Gambaro G, Emma F (2021). Distal renal tubular acidosis: a systematic approach from diagnosis to treatment. Journal of Nephrology.

[bib20] Heuser J (1989). Changes in lysosome shape and distribution correlated with changes in cytoplasmic pH. The Journal of Cell Biology.

[bib21] Höhne M, Frese CK, Grahammer F, Dafinger C, Ciarimboli G, Butt L, Binz J, Hackl MJ, Rahmatollahi M, Kann M, Schneider S, Altintas MM, Schermer B, Reinheckel T, Göbel H, Reiser J, Huber TB, Kramann R, Seeger-Nukpezah T, Liebau MC, Beck BB, Benzing T, Beyer A, Rinschen MM (2018). Single-nephron proteomes connect morphology and function in proteinuric kidney disease. Kidney International.

[bib22] Hu M, Zhou N, Cai W, Xu H (2022). Lysosomal solute and water transport. The Journal of Cell Biology.

[bib23] Huang X, Yao J, Liu L, Chen J, Mei L, Huangfu J, Luo D, Wang X, Lin C, Chen X, Yang Y, Ouyang S, Wei F, Wang Z, Zhang S, Xiang T, Neculai D, Sun Q, Kong E, Tate EW, Yang A (2023). S-acylation of p62 promotes p62 droplet recruitment into autophagosomes in mammalian autophagy. Molecular Cell.

[bib24] Jonckheere AI, Smeitink JAM, Rodenburg RJT (2012). Mitochondrial ATP synthase: architecture, function and pathology. Journal of Inherited Metabolic Disease.

[bib25] Keskanokwong T, Shandro HJ, Johnson DE, Kittanakom S, Vilas GL, Thorner P, Reithmeier RAF, Akkarapatumwong V, Yenchitsomanus P, Casey JR (2007). Interaction of integrin-linked kinase with the kidney chloride/bicarbonate exchanger, kAE1. The Journal of Biological Chemistry.

[bib26] Khositseth S, Bruce LJ, Walsh SB, Bawazir WM, Ogle GD, Unwin RJ, Thong M-K, Sinha R, Choo KE, Chartapisak W, Kingwatanakul P, Sumboonnanonda A, Vasuvattakul S, Yenchitsomanus P, Wrong O (2012). Tropical distal renal tubular acidosis: clinical and epidemiological studies in 78 patients. QJM.

[bib27] Kimura S, Noda T, Yoshimori T (2007). Dissection of the autophagosome maturation process by a novel reporter protein, tandem fluorescent-tagged LC3. Autophagy.

[bib28] Klionsky DJ, Abdalla FC, Abeliovich H, Abraham RT, Acevedo-Arozena A, Adeli K, Agholme L, Agnello M, Agostinis P, Aguirre-Ghiso JA, Ahn HJ, Ait-Mohamed O, Ait-Si-Ali S, Akematsu T, Akira S, Al-Younes HM, Al-Zeer MA, Albert ML, Albin RL, Alegre-Abarrategui J, Aleo MF, Alirezaei M, Almasan A, Almonte-Becerril M, Amano A, Amaravadi R, Amarnath S, Amer AO, Andrieu-Abadie N, Anantharam V, Ann DK, Anoopkumar-Dukie S, Aoki H, Apostolova N, Arancia G, Aris JP, Asanuma K, Asare NYO, Ashida H, Askanas V, Askew DS, Auberger P, Baba M, Backues SK, Baehrecke EH, Bahr BA, Bai XY, Bailly Y, Baiocchi R, Baldini G, Balduini W, Ballabio A, Bamber BA, Bampton ETW, Bánhegyi G, Bartholomew CR, Bassham DC, Bast RC, Batoko H, Bay BH, Beau I, Béchet DM, Begley TJ, Behl C, Behrends C, Bekri S, Bellaire B, Bendall LJ, Benetti L, Berliocchi L, Bernardi H, Bernassola F, Besteiro S, Bhatia-Kissova I, Bi X, Biard-Piechaczyk M, Blum JS, Boise LH, Bonaldo P, Boone DL, Bornhauser BC, Bortoluci KR, Bossis I, Bost F, Bourquin JP, Boya P, Boyer-Guittaut M, Bozhkov PV, Brady NR, Brancolini C, Brech A, Brenman JE, Brennand A, Bresnick EH, Brest P, Bridges D, Bristol ML, Brookes PS, Brown EJ, Brumell JH, Brunetti-Pierri N, Brunk UT, Bulman DE, Bultman SJ, Bultynck G, Burbulla LF, Bursch W, Butchar JP, Buzgariu W, Bydlowski SP, Cadwell K, Cahová M, Cai D, Cai J, Cai Q, Calabretta B, Calvo-Garrido J, Camougrand N, Campanella M, Campos-Salinas J, Candi E, Cao L, Caplan AB, Carding SR, Cardoso SM, Carew JS, Carlin CR, Carmignac V, Carneiro LAM, Carra S, Caruso RA, Casari G, Casas C, Castino R, Cebollero E, Cecconi F, Celli J, Chaachouay H, Chae HJ, Chai CY, Chan DC, Chan EY, Chang RCC, Che CM, Chen CC, Chen GC, Chen GQ, Chen M, Chen Q, Chen SSL, Chen W, Chen X, Chen X, Chen X, Chen YG, Chen Y, Chen Y, Chen YJ, Chen Z, Cheng A, Cheng CHK, Cheng Y, Cheong H, Cheong JH, Cherry S, Chess-Williams R, Cheung ZH, Chevet E, Chiang HL, Chiarelli R, Chiba T, Chin LS, Chiou SH, Chisari FV, Cho CH, Cho DH, Choi AMK, Choi D, Choi KS, Choi ME, Chouaib S, Choubey D, Choubey V, Chu CT, Chuang TH, Chueh SH, Chun T, Chwae YJ, Chye ML, Ciarcia R, Ciriolo MR, Clague MJ, Clark RSB, Clarke PGH, Clarke R, Codogno P, Coller HA, Colombo MI, Comincini S, Condello M, Condorelli F, Cookson MR, Coombs GH, Coppens I, Corbalan R, Cossart P, Costelli P, Costes S, Coto-Montes A, Couve E, Coxon FP, Cregg JM, Crespo JL, Cronjé MJ, Cuervo AM, Cullen JJ, Czaja MJ, D’Amelio M, Darfeuille-Michaud A, Davids LM, Davies FE, De Felici M, de Groot JF, de Haan CAM, De Martino L, De Milito A, De Tata V, Debnath J, Degterev A, Dehay B, Delbridge LMD, Demarchi F, Deng YZ, Dengjel J, Dent P, Denton D, Deretic V, Desai SD, Devenish RJ, Di Gioacchino M, Di Paolo G, Di Pietro C, Díaz-Araya G, Díaz-Laviada I, Diaz-Meco MT, Diaz-Nido J, Dikic I, Dinesh-Kumar SP, Ding WX, Distelhorst CW, Diwan A, Djavaheri-Mergny M, Dokudovskaya S, Dong Z, Dorsey FC, Dosenko V, Dowling JJ, Doxsey S, Dreux M, Drew ME, Duan Q, Duchosal MA, Duff K, Dugail I, Durbeej M, Duszenko M, Edelstein CL, Edinger AL, Egea G, Eichinger L, Eissa NT, Ekmekcioglu S, El-Deiry WS, Elazar Z, Elgendy M, Ellerby LM, Eng KE, Engelbrecht AM, Engelender S, Erenpreisa J, Escalante R, Esclatine A, Eskelinen EL, Espert L, Espina V, Fan H, Fan J, Fan QW, Fan Z, Fang S, Fang Y, Fanto M, Fanzani A, Farkas T, Farré JC, Faure M, Fechheimer M, Feng CG, Feng J, Feng Q, Feng Y, Fésüs L, Feuer R, Figueiredo-Pereira ME, Fimia GM, Fingar DC, Finkbeiner S, Finkel T, Finley KD, Fiorito F, Fisher EA, Fisher PB, Flajolet M, Florez-McClure ML, Florio S, Fon EA, Fornai F, Fortunato F, Fotedar R, Fowler DH, Fox HS, Franco R, Frankel LB, Fransen M, Fuentes JM, Fueyo J, Fujii J, Fujisaki K, Fujita E, Fukuda M, Furukawa RH, Gaestel M, Gailly P, Gajewska M, Galliot B, Galy V, Ganesh S, Ganetzky B, Ganley IG, Gao FB, Gao GF, Gao J, Garcia L, Garcia-Manero G, Garcia-Marcos M, Garmyn M, Gartel AL, Gatti E, Gautel M, Gawriluk TR, Gegg ME, Geng J, Germain M, Gestwicki JE, Gewirtz DA, Ghavami S, Ghosh P, Giammarioli AM, Giatromanolaki AN, Gibson SB, Gilkerson RW, Ginger ML, Ginsberg HN, Golab J, Goligorsky MS, Golstein P, Gomez-Manzano C, Goncu E, Gongora C, Gonzalez CD, Gonzalez R, González-Estévez C, González-Polo RA, Gonzalez-Rey E, Gorbunov NV, Gorski S, Goruppi S, Gottlieb RA, Gozuacik D, Granato GE, Grant GD, Green KN, Gregorc A, Gros F, Grose C, Grunt TW, Gual P, Guan JL, Guan KL, Guichard SM, Gukovskaya AS, Gukovsky I, Gunst J, Gustafsson AB, Halayko AJ, Hale AN, Halonen SK, Hamasaki M, Han F, Han T, Hancock MK, Hansen M, Harada H, Harada M, Hardt SE, Harper JW, Harris AL, Harris J, Harris SD, Hashimoto M, Haspel JA, Hayashi S, Hazelhurst LA, He C, He YW, Hébert MJ, Heidenreich KA, Helfrich MH, Helgason GV, Henske EP, Herman B, Herman PK, Hetz C, Hilfiker S, Hill JA, Hocking LJ, Hofman P, Hofmann TG, Höhfeld J, Holyoake TL, Hong MH, Hood DA, Hotamisligil GS, Houwerzijl EJ, Høyer-Hansen M, Hu B, Hu CAA, Hu HM, Hua Y, Huang C, Huang J, Huang S, Huang WP, Huber TB, Huh WK, Hung TH, Hupp TR, Hur GM, Hurley JB, Hussain SNA, Hussey PJ, Hwang JJ, Hwang S, Ichihara A, Ilkhanizadeh S, Inoki K, Into T, Iovane V, Iovanna JL, Ip NY, Isaka Y, Ishida H, Isidoro C, Isobe K, Iwasaki A, Izquierdo M, Izumi Y, Jaakkola PM, Jäättelä M, Jackson GR, Jackson WT, Janji B, Jendrach M, Jeon JH, Jeung EB, Jiang H, Jiang H, Jiang JX, Jiang M, Jiang Q, Jiang X, Jiang X, Jiménez A, Jin M, Jin S, Joe CO, Johansen T, Johnson DE, Johnson GVW, Jones NL, Joseph B, Joseph SK, Joubert AM, Juhász G, Juillerat-Jeanneret L, Jung CH, Jung YK, Kaarniranta K, Kaasik A, Kabuta T, Kadowaki M, Kagedal K, Kamada Y, Kaminskyy VO, Kampinga HH, Kanamori H, Kang C, Kang KB, Kang KI, Kang R, Kang YA, Kanki T, Kanneganti TD, Kanno H, Kanthasamy AG, Kanthasamy A, Karantza V, Kaushal GP, Kaushik S, Kawazoe Y, Ke PY, Kehrl JH, Kelekar A, Kerkhoff C, Kessel DH, Khalil H, Kiel J, Kiger AA, Kihara A, Kim DR, Kim DH, Kim DH, Kim EK, Kim HR, Kim JS, Kim JH, Kim JC, Kim JK, Kim PK, Kim SW, Kim YS, Kim Y, Kimchi A, Kimmelman AC, King JS, Kinsella TJ, Kirkin V, Kirshenbaum LA, Kitamoto K, Kitazato K, Klein L, Klimecki WT, Klucken J, Knecht E, Ko BCB, Koch JC, Koga H, Koh JY, Koh YH, Koike M, Komatsu M, Kominami E, Kong HJ, Kong WJ, Korolchuk VI, Kotake Y, Koukourakis MI, Kouri Flores JB, Kovács AL, Kraft C, Krainc D, Krämer H, Kretz-Remy C, Krichevsky AM, Kroemer G, Krüger R, Krut O, Ktistakis NT, Kuan CY, Kucharczyk R, Kumar A, Kumar R, Kumar S, Kundu M, Kung HJ, Kurz T, Kwon HJ, La Spada AR, Lafont F, Lamark T, Landry J, Lane JD, Lapaquette P, Laporte JF, László L, Lavandero S, Lavoie JN, Layfield R, Lazo PA, Le W, Le Cam L, Ledbetter DJ, Lee AJX, Lee BW, Lee GM, Lee J, Lee JH, Lee M, Lee MS, Lee SH, Leeuwenburgh C, Legembre P, Legouis R, Lehmann M, Lei HY, Lei QY, Leib DA, Leiro J, Lemasters JJ, Lemoine A, Lesniak MS, Lev D, Levenson VV, Levine B, Levy E, Li F, Li JL, Li L, Li S, Li W, Li XJ, Li Y, Li YP, Liang C, Liang Q, Liao YF, Liberski PP, Lieberman A, Lim HJ, Lim KL, Lim K, Lin CF, Lin FC, Lin J, Lin JD, Lin K, Lin WW, Lin WC, Lin YL, Linden R, Lingor P, Lippincott-Schwartz J, Lisanti MP, Liton PB, Liu B, Liu CF, Liu K, Liu L, Liu QA, Liu W, Liu YC, Liu Y, Lockshin RA, Lok CN, Lonial S, Loos B, Lopez-Berestein G, López-Otín C, Lossi L, Lotze MT, Lőw P, Lu B, Lu B, Lu B, Lu Z, Luciano F, Lukacs NW, Lund AH, Lynch-Day MA, Ma Y, Macian F, MacKeigan JP, Macleod KF, Madeo F, Maiuri L, Maiuri MC, Malagoli D, Malicdan MCV, Malorni W, Man N, Mandelkow EM, Manon S, Manov I, Mao K, Mao X, Mao Z, Marambaud P, Marazziti D, Marcel YL, Marchbank K, Marchetti P, Marciniak SJ, Marcondes M, Mardi M, Marfe G, Mariño G, Markaki M, Marten MR, Martin SJ, Martinand-Mari C, Martinet W, Martinez-Vicente M, Masini M, Matarrese P, Matsuo S, Matteoni R, Mayer A, Mazure NM, McConkey DJ, McConnell MJ, McDermott C, McDonald C, McInerney GM, McKenna SL, McLaughlin B, McLean PJ, McMaster CR, McQuibban GA, Meijer AJ, Meisler MH, Meléndez A, Melia TJ, Melino G, Mena MA, Menendez JA, Menna-Barreto RFS, Menon MB, Menzies FM, Mercer CA, Merighi A, Merry DE, Meschini S, Meyer CG, Meyer TF, Miao CY, Miao JY, Michels PAM, Michiels C, Mijaljica D, Milojkovic A, Minucci S, Miracco C, Miranti CK, Mitroulis I, Miyazawa K, Mizushima N, Mograbi B, Mohseni S, Molero X, Mollereau B, Mollinedo F, Momoi T, Monastyrska I, Monick MM, Monteiro MJ, Moore MN, Mora R, Moreau K, Moreira PI, Moriyasu Y, Moscat J, Mostowy S, Mottram JC, Motyl T, Moussa CEH, Müller S, Muller S, Münger K, Münz C, Murphy LO, Murphy ME, Musarò A, Mysorekar I, Nagata E, Nagata K, Nahimana A, Nair U, Nakagawa T, Nakahira K, Nakano H, Nakatogawa H, Nanjundan M, Naqvi NI, Narendra DP, Narita M, Navarro M, Nawrocki ST, Nazarko TY, Nemchenko A, Netea MG, Neufeld TP, Ney PA, Nezis IP, Nguyen HP, Nie D, Nishino I, Nislow C, Nixon RA, Noda T, Noegel AA, Nogalska A, Noguchi S, Notterpek L, Novak I, Nozaki T, Nukina N, Nürnberger T, Nyfeler B, Obara K, Oberley TD, Oddo S, Ogawa M, Ohashi T, Okamoto K, Oleinick NL, Oliver FJ, Olsen LJ, Olsson S, Opota O, Osborne TF, Ostrander GK, Otsu K, Ou JJ, Ouimet M, Overholtzer M, Ozpolat B, Paganetti P, Pagnini U, Pallet N, Palmer GE, Palumbo C, Pan T, Panaretakis T, Pandey UB, Papackova Z, Papassideri I, Paris I, Park J, Park OK, Parys JB, Parzych KR, Patschan S, Patterson C, Pattingre S, Pawelek JM, Peng J, Perlmutter DH, Perrotta I, Perry G, Pervaiz S, Peter M, Peters GJ, Petersen M, Petrovski G, Phang JM, Piacentini M, Pierre P, Pierrefite-Carle V, Pierron G, Pinkas-Kramarski R, Piras A, Piri N, Platanias LC, Pöggeler S, Poirot M, Poletti A, Poüs C, Pozuelo-Rubio M, Prætorius-Ibba M, Prasad A, Prescott M, Priault M, Produit-Zengaffinen N, Progulske-Fox A, Proikas-Cezanne T, Przedborski S, Przyklenk K, Puertollano R, Puyal J, Qian SB, Qin L, Qin ZH, Quaggin SE, Raben N, Rabinowich H, Rabkin SW, Rahman I, Rami A, Ramm G, Randall G, Randow F, Rao VA, Rathmell JC, Ravikumar B, Ray SK, Reed BH, Reed JC, Reggiori F, Régnier-Vigouroux A, Reichert AS, Reiners JJ, Reiter RJ, Ren J, Revuelta JL, Rhodes CJ, Ritis K, Rizzo E, Robbins J, Roberge M, Roca H, Roccheri MC, Rocchi S, Rodemann HP, Rodríguez de Córdoba S, Rohrer B, Roninson IB, Rosen K, Rost-Roszkowska MM, Rouis M, Rouschop KMA, Rovetta F, Rubin BP, Rubinsztein DC, Ruckdeschel K, Rudich A, Rucker EB, Rudolf E, Ruiz-Opazo N, Russo R, Rusten TE, Ryan KM, Ryter SW, Sabatini DM, Sadoshima J, Saha T, Saitoh T, Sakagami H, Sakai Y, Salekdeh GH, Salomoni P, Salvaterra PM, Salvesen G, Salvioli R, Sanchez AMJ, Sánchez-Alcázar JA, Sánchez-Prieto R, Sandri M, Sankar U, Sansanwal P, Santambrogio L, Saran S, Sarkar S, Sarwal M, Sasakawa C, Sasnauskiene A, Sass M, Sato K, Sato M, Schapira AHV, Scharl M, Schätzl HM, Scheper W, Schiaffino S, Schneider C, Schneider ME, Schneider-Stock R, Schoenlein PV, Schorderet DF, Schüller C, Schwartz GK, Scorrano L, Sealy L, Seglen PO, Segura-Aguilar J, Seiliez I, Seleverstov O, Sell C, Seo JB, Separovic D, Setaluri V, Setoguchi T, Settembre C, Shacka JJ, Shanmugam M, Shapiro IM, Shaulian E, Shaw RJ, Shelhamer JH, Shen HM, Shen WC, Sheng ZH, Shi Y, Shibuya K, Shidoji Y, Shieh JJ, Shih CM, Shimada Y, Shimizu S, Shintani T, Shirihai OS, Shore GC, Sibirny AA, Sidhu SB, Sikorska B, Silva-Zacarin ECM, Simmons A, Simon AK, Simon HU, Simone C, Simonsen A, Sinclair DA, Singh R, Sinha D, Sinicrope FA, Sirko A, Siu PM, Sivridis E, Skop V, Skulachev VP, Slack RS, Smaili SS, Smith DR, Soengas MS, Soldati T, Song X, Sood AK, Soong TW, Sotgia F, Spector SA, Spies CD, Springer W, Srinivasula SM, Stefanis L, Steffan JS, Stendel R, Stenmark H, Stephanou A, Stern ST, Sternberg C, Stork B, Strålfors P, Subauste CS, Sui X, Sulzer D, Sun J, Sun SY, Sun ZJ, Sung JJY, Suzuki K, Suzuki T, Swanson MS, Swanton C, Sweeney ST, Sy LK, Szabadkai G, Tabas I, Taegtmeyer H, Tafani M, Takács-Vellai K, Takano Y, Takegawa K, Takemura G, Takeshita F, Talbot NJ, Tan KSW, Tanaka K, Tanaka K, Tang D, Tang D, Tanida I, Tannous BA, Tavernarakis N, Taylor GS, Taylor GA, Taylor JP, Terada LS, Terman A, Tettamanti G, Thevissen K, Thompson CB, Thorburn A, Thumm M, Tian F, Tian Y, Tocchini-Valentini G, Tolkovsky AM, Tomino Y, Tönges L, Tooze SA, Tournier C, Tower J, Towns R, Trajkovic V, Travassos LH, Tsai TF, Tschan MP, Tsubata T, Tsung A, Turk B, Turner LS, Tyagi SC, Uchiyama Y, Ueno T, Umekawa M, Umemiya-Shirafuji R, Unni VK, Vaccaro MI, Valente EM, Van den Berghe G, van der Klei IJ, van Doorn W, van Dyk LF, van Egmond M, van Grunsven LA, Vandenabeele P, Vandenberghe WP, Vanhorebeek I, Vaquero EC, Velasco G, Vellai T, Vicencio JM, Vierstra RD, Vila M, Vindis C, Viola G, Viscomi MT, Voitsekhovskaja OV, von Haefen C, Votruba M, Wada K, Wade-Martins R, Walker CL, Walsh CM, Walter J, Wan XB, Wang A, Wang C, Wang D, Wang F, Wang F, Wang G, Wang H, Wang HG, Wang HD, Wang J, Wang K, Wang M, Wang RC, Wang X, Wang X, Wang YJ, Wang Y, Wang Z, Wang ZC, Wang Z, Wansink DG, Ward DM, Watada H, Waters SL, Webster P, Wei L, Weihl CC, Weiss WA, Welford SM, Wen LP, Whitehouse CA, Whitton JL, Whitworth AJ, Wileman T, Wiley JW, Wilkinson S, Willbold D, Williams RL, Williamson PR, Wouters BG, Wu C, Wu DC, Wu WKK, Wyttenbach A, Xavier RJ, Xi Z, Xia P, Xiao G, Xie Z, Xie Z, Xu D, Xu J, Xu L, Xu X, Yamamoto A, Yamamoto A, Yamashina S, Yamashita M, Yan X, Yanagida M, Yang DS, Yang E, Yang JM, Yang SY, Yang W, Yang WY, Yang Z, Yao MC, Yao TP, Yeganeh B, Yen WL, Yin J, Yin XM, Yoo OJ, Yoon G, Yoon SY, Yorimitsu T, Yoshikawa Y, Yoshimori T, Yoshimoto K, You HJ, Youle RJ, Younes A, Yu L, Yu L, Yu SW, Yu WH, Yuan ZM, Yue Z, Yun CH, Yuzaki M, Zabirnyk O, Silva-Zacarin E, Zacks D, Zacksenhaus E, Zaffaroni N, Zakeri Z, Zeitlin SO, Zeh HJ, Zhang H, Zhang HL, Zhang J, Zhang JP, Zhang L, Zhang L, Zhang MY, Zhang XD, Zhao M, Zhao YF, Zhao Y, Zhao ZJ, Zheng X, Zhivotovsky B, Zhong Q, Zhou CZ, Zhu C, Zhu WG, Zhu XF, Zhu X, Zhu Y, Zoladek T, Zong WX, Zorzano A, Zschocke J, Zuckerbraun B (2012). Guidelines for the use and interpretation of assays for monitoring autophagy. Autophagy.

[bib29] Kollert-Jons A, Wagner S, Hubner S, Appelhans H, Drenckhahn D (1993). Anion exchanger 1 in human kidney and oncocytoma differs from erythroid AE1 in its NH2 terminus. American Journal of Physiology-Renal Physiology.

[bib30] Korolchuk VI, Saiki S, Lichtenberg M, Siddiqi FH, Roberts EA, Imarisio S, Jahreiss L, Sarkar S, Futter M, Menzies FM, O’Kane CJ, Deretic V, Rubinsztein DC (2011). Lysosomal positioning coordinates cellular nutrient responses. Nature Cell Biology.

[bib31] Lashhab R, Rumley AC, Arutyunov D, Rizvi M, You C, Dimke H, Touret N, Zimmermann R, Jung M, Chen X-Z, Alexander T, Cordat E (2019). The kidney anion exchanger 1 affects tight junction properties via claudin-4. Scientific Reports.

[bib32] Lee WK, Probst S, Santoyo-Sánchez MP, Al-Hamdani W, Diebels I, von Sivers JK, Kerek E, Prenner EJ, Thévenod F (2017). Initial autophagic protection switches to disruption of autophagic flux by lysosomal instability during cadmium stress accrual in renal NRK-52E cells. Archives of Toxicology.

[bib33] Liu F, Zhou T, Zhang S, Li Y, Chen Y, Miao Z, Wang X, Yang G, Li Q, Zhang L, Liu Y (2024). Cathepsin B: The dawn of tumor therapy. European Journal of Medicinal Chemistry.

[bib34] Lyons JC, Kim GE, Song CW (1992). Modification of intracellular pH and thermosensitivity. Radiation Research.

[bib35] Mizushima N, Ohsumi Y, Yoshimori T (2002). Autophagosome formation in mammalian cells. Cell Structure and Function.

[bib36] Mizushima N, Komatsu M (2011). Autophagy: renovation of cells and tissues. Cell.

[bib37] Mizushima N (2018). A brief history of autophagy from cell biology to physiology and disease. Nature Cell Biology.

[bib38] Mumtaz R, Trepiccione F, Hennings JC, Huebner AK, Serbin B, Picard N, Ullah A, Păunescu TG, Capen DE, Lashhab RM, Mouro-Chanteloup I, Alper SL, Wagner CA, Cordat E, Brown D, Eladari D, Hübner CA (2017). Intercalated cell depletion and vacuolar H^+^-ATPase Mistargeting in an Ae1 R607H Knockin Model. Journal of the American Society of Nephrology.

[bib39] Mungara P, MacNaughton K, Ullah AS, Essuman G, Chelangarimiyandoab F, Mumtaz R, Hennings JC, Hübner CA, Eladari D, Alexander RT, Cordat E (2024). Urinary sodium wasting and disrupted collecting duct function in mice with dRTA-Causing *SLC4A1* Mutations. bioRxiv.

[bib40] Mungara P, MacNaughton K, Ullah AKMS, Essuman G, Chelangarimiyandoab F, Mumtaz R, Hennings JC, Hübner CA, Eladari D, Alexander RT, Cordat E (2025). Urinary sodium wasting and disrupted collecting duct function in mice with distal renal tubular acidosis mutations. Disease Models & Mechanisms.

[bib41] Namba T, Takabatake Y, Kimura T, Takahashi A, Yamamoto T, Matsuda J, Kitamura H, Niimura F, Matsusaka T, Iwatani H, Matsui I, Kaimori J, Kioka H, Isaka Y, Rakugi H (2014). Autophagic clearance of mitochondria in the kidney copes with metabolic acidosis. Journal of the American Society of Nephrology.

[bib42] Nguyen TT, Quan X, Xu S, Das R, Cha S-K, Kong ID, Shong M, Wollheim CB, Park K-S (2016). Intracellular alkalinization by phosphate uptake via type III sodium-phosphate cotransporter participates in high-phosphate-induced mitochondrial oxidative stress and defective insulin secretion. FASEB Journal.

[bib43] Nuiplot N-O, Junking M, Duangtum N, Khunchai S, Sawasdee N, Yenchitsomanus P-T, Akkarapatumwong V (2015). Transmembrane protein 139 (TMEM139) interacts with human kidney isoform of anion exchanger 1 (kAE1). Biochemical and Biophysical Research Communications.

[bib44] Ratto E, Chowdhury SR, Siefert NS, Schneider M, Wittmann M, Helm D, Palm W (2022). Direct control of lysosomal catabolic activity by mTORC1 through regulation of V-ATPase assembly. Nature Communications.

[bib45] Ribeiro ML, Alloisio N, Almeida H, Gomes C, Texier P, Lemos C, Mimoso G, Morlé L, Bey-Cabet F, Rudigoz RC, Delaunay J, Tamagnini G (2000). Severe hereditary spherocytosis and distal renal tubular acidosis associated with the total absence of band 3. Blood.

[bib46] Romero MF, Chen AP, Parker MD, Boron WF (2013). The SLC4 family of bicarbonate (HCO_3_⁻) transporters. Molecular Aspects of Medicine.

[bib47] Rungroj N, Nettuwakul C, Sawasdee N, Sangnual S, Deejai N, Misgar RA, Pasena A, Khositseth S, Kirdpon S, Sritippayawan S, Vasuvattakul S, Yenchitsomanus PT (2018). Distal renal tubular acidosis caused by tryptophan-aspartate repeat domain 72 (WDR72) mutations. Clinical Genetics.

[bib48] Sawasdee N, Udomchaiprasertkul W, Noisakran S, Rungroj N, Akkarapatumwong V, Yenchitsomanus P (2006). Trafficking defect of mutant kidney anion exchanger 1 (kAE1) proteins associated with distal renal tubular acidosis and Southeast Asian ovalocytosis. Biochemical and Biophysical Research Communications.

[bib49] Sawasdee N, Junking M, Ngaojanlar P, Sukomon N, Ungsupravate D, Limjindaporn T, Akkarapatumwong V, Noisakran S, Yenchitsomanus P-T (2010). Human kidney anion exchanger 1 interacts with adaptor-related protein complex 1 μ1A (AP-1 mu1A). Biochemical and Biophysical Research Communications.

[bib50] Saxena V, Gao H, Arregui S, Zollman A, Kamocka MM, Xuei X, McGuire P, Hutchens M, Hato T, Hains DS, Schwaderer AL (2021). Kidney intercalated cells are phagocytic and acidify internalized uropathogenic *Escherichia coli*. Nature Communications.

[bib51] Schindelin J, Arganda-Carreras I, Frise E, Kaynig V, Longair M, Pietzsch T, Preibisch S, Rueden C, Saalfeld S, Schmid B, Tinevez J-Y, White DJ, Hartenstein V, Eliceiri K, Tomancak P, Cardona A (2012). Fiji: an open-source platform for biological-image analysis. Nature Methods.

[bib52] Schütt F, Aretz S, Auffarth GU, Kopitz J (2012). Moderately reduced ATP levels promote oxidative stress and debilitate autophagic and phagocytic capacities in human RPE cells. Investigative Ophthalmology & Visual Science.

[bib53] Sorrell SL, Golder ZJ, Johnstone DB, Frankl FEK (2016). Renal peroxiredoxin 6 interacts with anion exchanger 1 and plays a novel role in pH homeostasis. Kidney International.

[bib54] Sterling D, Casey JR (1999). Transport activity of AE3 chloride/bicarbonate anion-exchange proteins and their regulation by intracellular pH. The Biochemical Journal.

[bib55] Stirling DR, Swain-Bowden MJ, Lucas AM, Carpenter AE, Cimini BA, Goodman A (2021). CellProfiler 4: improvements in speed, utility and usability. BMC Bioinformatics.

[bib56] Su Y, Blake-Palmer KG, Fry AC, Best A, Brown ACN, Hiemstra TF, Horita S, Zhou A, Toye AM, Karet FE (2011). Glyceraldehyde 3-phosphate dehydrogenase is required for band 3 (anion exchanger 1) membrane residency in the mammalian kidney. American Journal of Physiology. Renal Physiology.

[bib57] Tang X, Guo X, Gao J (2020). A novel compound heterozygous mutation in SLC4A1 gene causing severe hereditary spherocytosis and distal renal tubular acidosis. Indian Journal of Pediatrics.

[bib58] Thornell IM, Bevensee MO (2015). Regulators of Slc4 bicarbonate transporter activity. Frontiers in Physiology.

[bib59] Toye AM, Banting G, Tanner MJA (2004). Regions of human kidney anion exchanger 1 (kAE1) required for basolateral targeting of kAE1 in polarised kidney cells: mis-targeting explains dominant renal tubular acidosis (dRTA). Journal of Cell Science.

[bib60] Toye AM, Williamson RC, Khanfar M, Bader-Meunier B, Cynober T, Thibault M, Tchernia G, Déchaux M, Delaunay J, Bruce LJ (2008). Band 3 Courcouronnes (Ser667Phe): a trafficking mutant differentially rescued by wild-type band 3 and glycophorin A. Blood.

[bib61] Wu F, Saleem MA, Kampik NB, Satchwell TJ, Williamson RC, Blattner SM, Ni L, Toth T, White G, Young MT, Parker MD, Alper SL, Wagner CA, Toye AM (2010). Anion exchanger 1 interacts with nephrin in podocytes. Journal of the American Society of Nephrology.

[bib62] Xu T, Su H, Ganapathy S, Yuan ZM (2011). Modulation of autophagic activity by extracellular pH. Autophagy.

[bib63] Yamamoto T, Takabatake Y, Takahashi A, Kimura T, Namba T, Matsuda J, Minami S, Kaimori J-Y, Matsui I, Matsusaka T, Niimura F, Yanagita M, Isaka Y (2017). High-fat diet-induced lysosomal dysfunction and impaired autophagic flux contribute to lipotoxicity in the kidney. Journal of the American Society of Nephrology.

[bib64] Yang M, Sheng Q, Ge S, Song X, Dong J, Guo C, Liao L (2023). Mutations and clinical characteristics of dRTA caused by *SLC4A1* mutations: Analysis based on published patients. Frontiers in Pediatrics.

[bib65] Zhang Q, Jian L, Yao D, Rao B, Xia Y, Hu K, Li S, Shen Y, Cao M, Qin A, Zhao J, Cao Y (2023). The structural basis of the pH-homeostasis mediated by the Cl−/HCO3− exchanger, AE2. Nature Communications.

[bib66] Zhou C, Zhong W, Zhou J, Sheng F, Fang Z, Wei Y, Chen Y, Deng X, Xia B, Lin J (2012). Monitoring autophagic flux by an improved tandem fluorescent-tagged LC3 (mTagRFP-mWasabi-LC3) reveals that high-dose rapamycin impairs autophagic flux in cancer cells. Autophagy.

